# The relationship between newspaper reading preferences and attitudes towards autism

**DOI:** 10.1177/13623613251394523

**Published:** 2025-12-03

**Authors:** Marta Dickinson, Themis Karaminis

**Affiliations:** 1 Edge Hill University, UK; 2 City St George’s, University of London, UK

**Keywords:** attitudes, autism, explicit, implicit, media influence, newspapers, stigma

## Abstract

Newspapers – particularly certain tabloid and right-leaning publications – often perpetuate negative stereotypes of Autistic people. This study examined how newspaper reading preferences relate to public attitudes towards autism. A sample of 277 UK-based non-autistic adults completed an online survey reporting demographic information, newspaper reading habits (print or online) and trustworthiness ratings for 10 major British newspapers. Participants also completed measures of autism-related knowledge and explicit attitudes towards autism, and an implicit bias task. Data were analysed using generalized additive models weighted by newspaper exposure, along with hierarchical partitioning to estimate the variance explained by reading preferences and other factors. Newspaper-related factors accounted for 4.5% of the variance in explicit attitudes and 10.7% in implicit attitudes. Crucially, selective engagement with right-leaning tabloids was associated with more negative implicit attitudes. Furthermore, selective trust in these outlets predicted relatively favourable explicit but relatively negative implicit attitudes. Overall trust in newspapers was also linked to less accurate autism knowledge. These findings suggest that engagement with media sources portraying autism more negatively is linked to more negative implicit biases towards Autistic people, even when explicit attitudes remain relatively favourable. Future research should explore causal mechanisms and the broader influence of media ecosystems on public perceptions of autism.

**Lay abstract:**

When newspapers discuss Autistic people, they often focus on their challenges rather than their strengths. This kind of reporting – especially in some tabloids and right-leaning newspapers – can reinforce negative stereotypes, making it harder to build a more inclusive society for Autistic people. However, we do not yet fully understand how newspaper coverage relates to neurotypical people’s attitudes towards autism, particularly when considering their background, knowledge of autism and personal experiences with Autistic people. This study investigated whether there is a connection between the newspapers people read and trust, and their feelings about autism. We examined both openly expressed opinions (explicit attitudes) and more instinctive, less conscious reactions (implicit attitudes). We surveyed 277 non-autistic adults in the United Kingdom. Participants reported how often they read 10 major British newspapers (in print or online) and how much they trusted them. They also answered questions about their knowledge of autism and their attitudes towards Autistic people. In addition, participants completed a short word-based task designed to reveal more subtle, instinctive responses. The results showed that individuals who regularly read right-leaning tabloids – which more frequently feature negative coverage of autism – tended to display more negative automatic responses towards autism. Interestingly, some participants who highly trusted these outlets expressed relatively positive explicit views, while their task responses suggested they might still hold relatively negative unconscious biases. Finally, greater overall trust in newspapers was linked to lower levels of autism knowledge. Taken together, these findings highlight a potential relationship between the media we consume and trust and not only what we know, believe and openly say about autism, but also our deeper, less conscious attitudes and reactions. While this study does not prove that news media directly shape or cause changes in attitudes, it underscores the importance of respectful, balanced reporting in fostering greater understanding and acceptance of Autistic people.

## Introduction

Autistic people often face persistent challenges and adverse outcomes in education, employment, social relationships and physical and mental health. Increasingly, researchers and advocates argue that these outcomes are shaped less by being Autistic and more by insufficient environmental accommodations and broader societal exclusion (Botha & Frost, 2020; Pellicano & den Houting, 2022). Although the mechanisms underpinning the acceptance of autism are complex, prior research highlights associations between both individual characteristics (e.g. age, gender, personal experience) and environmental factors (e.g. culture) and laypeople’s attitudes towards Autistic people (Kim et al., 2023b). This study focuses on another environmental factor: media representations of autism, particularly in newspapers. It explores how newspaper reading preferences relate to attitudes towards autism, alongside a range of other established factors. Using methods that estimate the individual contributions of these factors, the study seeks to advance our understanding of how media portrayals may be associated with public perceptions of autism.

### The role of acceptance in autism-related adversities

Autistic people, who comprise approximately 1%–3% of the population (Zeidan et al., 2022), face challenges and disadvantages across the lifespan – from increased bullying and school exclusion (Brede et al., 2017; Danker et al., 2019; Schroeder et al., 2014), to social isolation at university (Lipson et al., 2020; Sasson et al., 2017), and reduced employment outcomes (Vincent & Fabri, 2022). Mental health conditions are also more prevalent among Autistic people (Charlton et al., 2020; Hollocks et al., 2019; M. Lai et al., 2019; Smith et al., 2019).

Historically, these difficulties were often attributed to presumed ‘deficits’ in communication or social interaction (American Psychiatric Association [APA], 2022; World Health Organization [WHO], 2022). More recent perspectives, however, emphasize the role of systemic barriers and unsupportive environments (Kapp et al., 2013; Pellicano & den Houting, 2022). While autism may involve disabling aspects, these are intensified by insufficient accommodations (Botha & Frost, 2020). Within this framework, many challenges Autistic people face are relational, that is, they emerge from interactions with a predominantly non-autistic and non-accepting society (Botha & Gillespie-Lynch, 2022). Low societal acceptance can reinforce stigma, stereotypes and exclusion, potentially impacting self-esteem and contributing to masking behaviours, which may have adverse effects on mental health (Hull et al., 2021; Turnock et al., 2022). These dynamics perpetuate a vicious cycle of further marginalization and adversity (Bishop-Fitzpatrick et al., 2017; Cage et al., 2018; Griffith et al., 2012; Haslam et al., 2005; Milton & Sims, 2016).

### Attitudes towards Autistic people and contributing factors

A fundamental component of the cycle of marginalization and adversity are the attitudes neurotypical people hold towards autism. Researchers often categorize such attitudes into explicit and implicit attitudes. Explicit attitudes are consciously shaped and reflect cultural norms (Wilson et al., 2000). In contrast, implicit attitudes are viewed as automatic and less accessible, often indicating ingrained biases that are difficult to control (Dickter et al., 2020; Dovidio et al., 1997). The degree to which implicit attitudes are unconscious – and the ways in which this unconsciousness operates – remains a topic of debate (Holroyd et al., 2017). Nevertheless, while explicit attitudes may present as supportive, implicit negative biases could still subtly influence behaviour (Dickter et al., 2020; Dovidio et al., 2002). Research commonly finds that implicit attitudes are less favourable than explicit ones (Dickter et al., 2020), though findings regarding their relationship are varied, with some studies reporting no association (Cage & Doyle, 2024; Dickter et al., 2020) and others indicating stronger connections (Cheng et al., 2025; Kim et al., 2023a).

Another important factor associated with attitudes is knowledge about autism, which can be gained through training, study or public awareness campaigns (Gillespie-Lynch et al., 2015; D. R. Jones et al., 2021; Someki et al., 2018). Many studies suggest a link between increased autism knowledge with more favourable explicit attitudes (e.g. Au & Lau, 2021; Cage & Doyle, 2024; Cheng et al., 2025; Kuzminski et al., 2019; Shand et al., 2020), although some research indicates no such relationship, particularly for implicit attitudes (Bast et al., 2020; D. R. Jones et al., 2021). This suggests that certain biases may persist despite increased awareness.

Environmental factors – such as culture and interpersonal interactions – are also associated with attitudes towards autism. Research indicates that individuals in Western cultures often report more positive attitudes compared to those in some Eastern or Asian contexts (Cheng et al., 2025; Gillespie-Lynch et al., 2019; Keating et al., 2024; Kim et al., 2023a). Cross-cultural differences may be, partially, explained by cultural orientations and political values, such as the emphasis on individualism or collectivism, as well as the acceptance of inequality (Cheng et al., 2025; Gillespie-Lynch et al., 2021).

Contact with Autistic people is another key factor. Positive interactions are associated with reduced prejudice and improved attitudes, both explicit and implicit (Dickter & Burk, 2021; Gardiner & Iarocci, 2014; Nevill & White, 2011). The quality of contact is important, as sustained, constructive engagement is associated with greater empathy and understanding, while superficial or negative encounters may reinforce stereotypes (Gardiner & Iarocci, 2014; Gillespie-Lynch et al., 2019; McManus et al., 2011).

### Representation of Autistic people in news media

An alternative, relatively underexplored, environmental factor associated with attitudes towards autism is media representation, including fictional media, news coverage as well as social media (Draaisma, 2009; Jawed et al., 2023; S. C. Jones et al., 2023; Mittmann et al., 2024; Nordahl-Hansen et al., 2018). News media, in particular, are thought to reflect and shape public perceptions through agenda-setting, language and framing (Corrigan et al., 2005; Edelman, 1988; Happer & Philo, 2013). Several studies have indicated that newspapers often perpetuate ableist stereotypes and feature skewed representations of autism, for example, they focus on autistic children – especially boys (Akhtar et al., 2022; Baroutsis et al., 2023; Holton et al., 2014; Huws & Jones, 2011; Karaminis et al., 2023, 2025; Mittmann et al., 2024).

A large-scale study, conducted by Karaminis et al. (2023), systematically analysed autism representations in 10 British newspapers over a decade, tracking changes over time and differences between different outlets. The study revealed an increase in newspaper coverage of autism in the United Kingdom, accompanied by a gradual shift from deficit-focused to difference-oriented perspectives. However, this shift was less pronounced in tabloids and right-leaning publications, where references to autism remained less frequent and more negative or deficit-based. Furthermore, tabloid coverage often emphasized individual cases and celebrities associated with autism rather than providing nuanced, non-stigmatizing accounts of the autistic experience.

While there is a growing body of research on autism representation in news media, the extent to which these portrayals influence public understanding and attitudes towards Autistic people remains underexplored. Theories of media influence suggest that media and press coverage can affect public attitudes, often proportionally with the level of exposure (Arendt, 2010; Gerbner et al., 2002; Happer & Philo, 2013; Jamieson & Waldman, 2003). For example, the cultivation theory (Gerbner et al., 2002) posits that the long-term and repeated exposure to particular media portrayals may subtly shape the audience’s perceptions of social realities, and their attitudes and behaviours. However, the relationship between media representation and public opinion is complex and bidirectional, as public perceptions can both shape and be shaped by media coverage. Audiences often filter information through their existing beliefs and seek out sources that align with their perspectives (Arendt, 2010; Gerbner et al., 2002; Minson & Dorison, 2022). This phenomenon can be amplified by ‘echo chambers’, which reinforce existing biases by exposing individuals primarily to views that confirm their beliefs (Flaxman et al., 2016).

Finally, individual differences likely play an important role in how press representations influence attitudes. Demographic factors, prior personal contact with Autistic people and knowledge about autism may influence how non-autistic people perceive and interpret news stories about autism or Autistic people. Different groups may prefer certain news outlets or respond differently to coverage based on their beliefs and familiarity with autism.

### The current study

This study takes an initial step in examining how newspaper reading preferences might relate to explicit and implicit attitudes towards autism. Using an online survey, we recorded participants’ newspaper reading habits, trust in specific newspapers and their explicit and implicit attitudes towards Autistic people. We also recorded additional variables previously shown to be related with attitudes towards autism, such as age, gender, political views, contact with Autistic people and autism knowledge (Kim et al., 2023b).

We hypothesized that attitudes towards autism should vary based on reading behaviours and trust preferences, particularly between readers of right-leaning tabloids and left-leaning broadsheets. These groups represent opposite ends of two dimensions, namely, reporting style and political orientation, which have been shown to differ considerably in their portrayal of autism (Karaminis et al., 2023). Furthermore, we expected that factors such as age, gender, contact with Autistic people and autism knowledge would be associated with attitudes, in line with previous research (Kim et al., 2023b).

We acknowledge that the cross-sectional design of this study limits the ability to draw causal inferences from our data. Furthermore, the inclusion of multiple potentially interrelated factors complicates interpretation. While we refrain from making causal claims, we addressed the complexity arising from the simultaneous study of multiple factors through statistical means. Specifically, we used a recently proposed technique (referred to as hierarchical partitioning; J. Lai et al., 2024; see Methods) to estimate the individual contribution of reading habits, trust in newspapers and other factors in explaining intra-individual variability in both explicit and implicit attitudes towards autism. Furthermore, we conducted a post hoc analysis to assess the extent to which newspaper reading preferences accounted for variance in autism knowledge, which consistently emerged as a key predictor of attitudes towards autism.

## Methods

### Participants

Participant demographics are shown in Table 1. A total of 277 participants (180 identified as female, 96 as male and 1 as other) completed the study. Participants were recruited via the Prolific platform (www.prolific.co) and received monetary compensation proportional to the time spent to complete the study. The Prolific platform’s pre-screening functionality ensured all participants were over 18, UK residents, non-autistic and had access to a computer with a physical keyboard (necessary for the implicit attitudes assessment).

**Table 1.** Participants’ demographics and characteristics.

Demographic or characteristic	Category	Counts	Percentage
Gender	Female	180	65.0
	Male	96	34.7
	Other	1	0.4
Age	18–24	24	8.7
	25–34	77	27.8
	35–44	71	25.6
	45–54	44	15.9
	55–64	14	5.1
	65+	3	1.1
Education	GCSE or lower	38	13.7
	A-Levels or equivalent	51	18.4
	Certificate, Diploma or Foundation	31	11.2
	Degree	122	44.0
	Bachelor’s Degree	29	10.5
	Master’s Degree	6	2.2
	PhD or Professional Doctorate	0	0
Political leaning	Left	54	19.5
	Left-leaning	97	35.0
	Centre	83	30.0
	Right-leaning	25	9.0
	Right	3	1.1
	Other	3	1.1
	Prefer not to say	12	4.3
Contact with Autistic people	‘No, I don’t know anyone who is Autistic.’	67	24.2
	‘Yes, I know someone who is Autistic, but I don’t spend time with them.’	65	23.5
	‘Yes, I know someone who is Autistic, but I don’t spend time with them.’	67	24.2
	‘Yes, I know someone who is Autistic and spend time with them often or regularly.’	78	28.2

An additional 45 participants started but did not complete the survey and were excluded from the analysis. We also excluded 21 participants who failed at least one of two attention-check questions included in the survey to enhance data quality (A. Jones et al., 2022).

### Survey

All participants completed an online survey via Qualtrics (www.qualtrics.com). The survey included the following six sections.

#### Demographics

Participants began by answering four demographic questions. They indicated their age group (six ranges: 18–24, 25–34, 35–44, 45–54, 55–64, 65 and older), gender, their highest completed education level (GCSE or lower, A-Levels or equivalent, Certificate, Diploma or Foundation Degree, Bachelor’s Degree, Master’s Degree or PhD/Professional Doctorate). In addition, participants indicated their political leanings, choosing from six options: ‘Left’, ‘Left-leaning’, ‘Centre’, ‘Right-leaning’, ‘Right’, ‘Other’ and ‘Prefer not to say’.

#### Contact with Autistic people

Participants reported their level of contact with Autistic people by responding to the question: ‘Do you know and regularly spend time with someone who is Autistic?’ The response options included: ‘No, I don’t know anyone who is Autistic’, ‘Yes, I know someone who is Autistic, but I don’t spend time with them’, ‘Yes, I know someone who is Autistic, but I only spend time with them infrequently’ and ‘Yes, I know someone who is Autistic and spend time with them often or regularly’.

#### Newspaper reading preferences and perceived trustworthiness

Next, the survey asked participants about their reading habits and perceptions of 10 prominent British newspapers (*Daily Express*, *Daily Mail*, *Daily Mirror*, *Daily Star*, *Daily Telegraph*, *The Guardian*, *The Independent*, *The Observer*, *The Sun* and *The Times*). For each newspaper, participants indicated their reading frequency – for either print or online editions – using five options: ‘Never’, ‘A few times a year’, ‘A few times a month’, ‘A few times a week’ and ‘Daily’. In addition, they rated the trustworthiness of each newspaper on a 5-point Likert-type scale, ranging from ‘Not trustworthy’ to ‘Very trustworthy’.

#### Single Category Implicit Association Test

Participants were then invited to ‘complete a word-based game in which [they should] assign words to categories’, specifically a Single Category Implicit Association Test (SC-IAT; Karpinski & Steinman, 2006). This computerized task, implemented within Qualtrics with the *iatgen* platform (http://iatgen.org) and the iatgen R package (Carpenter et al., 2019) assessed implicit attitudes towards autism using stimuli-words identical to Cage and Doyle (2024). The SC-IAT measured the speed with which participants categorized autism-related words as positive or negative. The task comprised two stages, counterbalanced across participants: in one stage, autism-related words (namely, ‘Autistic’, ‘Asperger’s’, ‘Spectrum’, ‘ASD’ and ‘Neurodivergent’) were paired with positive evaluations (e.g. ‘wonderful’, ‘friendly’ and ‘happy’); in the other, with negative (e.g. ‘horrible’, ‘angry’ and ‘tragic’). Response times indicated the strength and direction of implicit associations, with faster responses suggesting stronger associations. The resulting D-score quantified implicit attitudes: values from −2.00 to −0.16 were taken to indicate negative attitudes, −0.15 to +0.15 neutral and +0.16 to +2.00 positive attitudes (Cage & Doyle, 2024; Dickter et al., 2020; Karpinski & Steinman, 2006).

**Figure 1.**
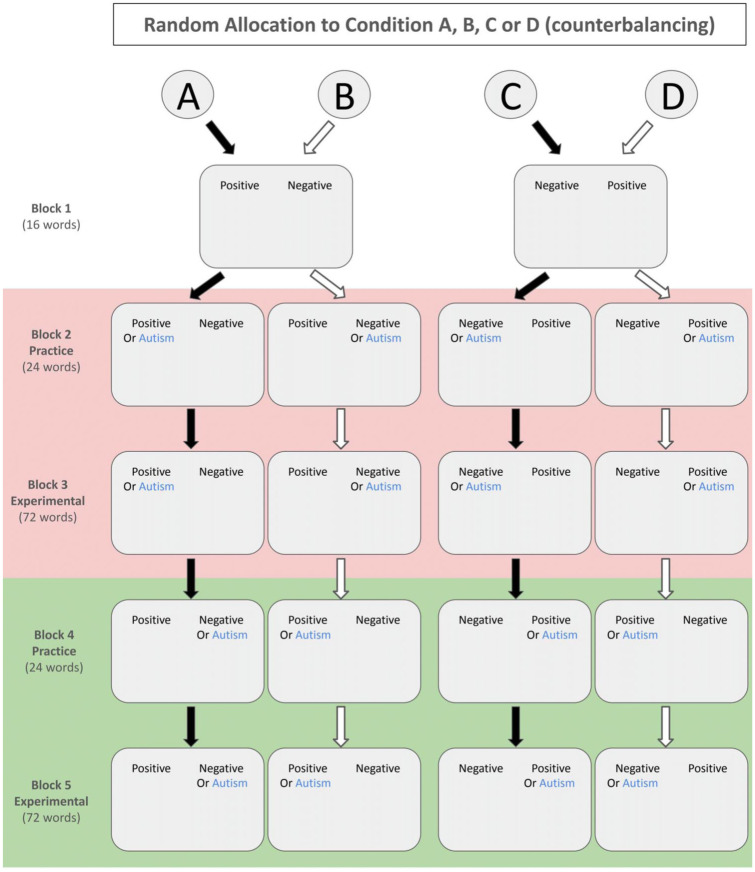
The structure of the Single Category Implicit Association Test (SC-IAT). The light red shading denotes the first stage of the task and the light green shading the second stage. Participants were randomly assigned to one of four counterbalancing conditions, which varied the position and order of blocks pairing autism-related words with either the positive or negative categories.

The structure of the SC-IAT is shown in Figure 1. Participants were assigned randomly to one of four experimental conditions (A–D) and completed five blocks of trials. The first block introduced the task and allowed participants to practice categorizing words. The second and third blocks formed the first stage of the SC-IAT, where participants categorized autism-related words with one evaluation. The fourth and fifth blocks constituted the second stage, where the opposite evaluation was applied. The between-block reliability was good in our data with Cronbach’s α = 0.82.

Block 1 comprised 16 practice trials and did not contribute to the D-score calculation. In this block, participants familiarized themselves with the task by using two specific keyboard keys (‘E’ and ‘I’) to categorize words with either positive or negative meanings. The labels ‘Positive’ and ‘Negative’ appeared at the top of the screen, randomly assigned to the left or right side. These labels remained in fixed positions throughout Block 1 to serve as reminders. We instructed participants that the ‘E’ key corresponded to the label on the left-hand side of the screen, whereas the ‘I’ key corresponded to the label on the right-hand side.

During each trial in Block 1, a word with either positive (e.g. ‘wonderful’, ‘excellent’) or negative (e.g. ‘tragic’, ‘dislike’) connotations appeared in the centre of the screen. Participants pressed the corresponding key to categorize each word as either positive or negative. For example, if ‘Positive’ was on the left side and ‘Negative’ on the right side of the screen, participants would press ‘E’ when the word ‘wonderful’ appeared. Incorrect responses triggered a red ‘X’, which remained in the centre of the screen until participants selected the correct response.

Participants then proceeded to Stage 1 of the SC-IAT, completing Blocks 2 (24 practice trials) and 3 (72 experimental trials). In these blocks, the phrase ‘OR Autism’ appeared beneath either the ‘Positive’ label (a ‘positive’ block in counterbalancing conditions A and B; see Figure 1) or the ‘Negative’ label (a ‘negative’ block in counterbalancing conditions 2 and 3; see Figure 1). This modification reminded participants to associate words related to the autism category with either a positive or negative evaluation, depending on their assigned condition.

Blocks 2 and 3 introduced words from the autism category (e.g. ‘Autistic’, ‘Asperger’s’) in the task alongside positive and negative words. All these words appeared, again, in the centre of the screen. Participants categorized each word using the ‘E’ and ‘I’ keys to indicate its appropriate classification (‘Autism’, ‘Positive’ or ‘Negative’). When participants should associate autism with ‘Positive’, the ratio of autism-related, positive and negative words was 7:7:10 (or 7:10:7 when participants associated autism with ‘Negative’). This configuration ensured a balanced ratio of correct responses using the ‘E’ and ‘I’ keys, resulting in a 58:42 (or 42:58) distribution of keypresses.

Stage 2 comprised Blocks 4 and 5, which mirrored Blocks 2 and 3 with 24 practice trials followed by 72 experimental trials. However, in Stage 2, the position of the ‘OR Autism’ label was opposite to that in Blocks 2 and 3, requiring participants to learn and apply a new pairing between the autism category and its corresponding evaluation.

#### The Societal Attitudes Towards Autism Scale

After the SC-IAT, participants completed the Societal Attitudes Towards Autism Scale (SATA; Flood et al., 2013), a 16-item questionnaire assessing explicit attitudes towards autism. The scale includes statements targeting stereotypes and biases (e.g. ‘People with autism should not engage in romantic relationships’), rated on a 4-point Likert-type scale. Higher scores on the SATA are generally interpreted as reflecting more positive attitudes towards autism; however, as noted by S. C. Jones (2022), it is possible for individuals who hold negative attitudes towards autism to nonetheless produce positive SATA scores (see also Cheng et al., 2025). SATA’s internal consistency in this study was good (Cronbach’s α = 0.84).

#### Autism Awareness Scale

Participants’ autism knowledge was measured using the Autism Awareness Scale (AAS; Gillespie-Lynch et al., 2015; Tipton & Blacher, 2014), comprising 13 items (e.g. ‘Autism is more frequently diagnosed in males than females’). Items were rated on a 5-point Likert-type scale. The AAS showed acceptable internal reliability (Cronbach’s α = 0.77).

### General procedure and ethics

Participants accessed the survey via Prolific, first reading an information sheet outlining the study’s focus on autism-related beliefs and everyday life (but without disclosing the focus on newspaper reading preferences). The study took approximately 15–20 min to complete. Two attention-check questions were embedded in the SATA and AAS scales (A. Jones et al., 2022). This research was approved by the Ethics Committee of Edge Hill University [Ref No: ETH2122-0341] and conducted in accordance with its ethical guidelines.

### Participatory methods

All stages of this quantitative survey study, including study design, data analysis, interpretation and manuscript preparation, were conducted collaboratively by an Autistic and a non-autistic researcher, who worked together within a supervisor–MSc dissertation student relationship during the 2022–2023 academic year. The process combined independent work with regular meetings that supported both the research and the course requirements.

The research questions and survey instruments were co-developed, with discussions aimed at ensuring both methodological rigour in the study of the relationship between reading preferences and attitudes, and relevance to autistic experiences. The survey also incorporated existing instruments, which the Autistic researcher evaluated in terms of their relevance; final decisions sought to balance methodological standards in earlier studies with sensitivity to autistic perspectives. The Autistic researcher further provided input on how survey items could be framed in ways that were accessible, respectful and meaningful to the autistic community.

Survey data were analysed jointly, with both researchers contributing to analytic decisions and collaboratively interpreting the findings. The Autistic researcher contributed insights informed by lived experience, while the non-autistic researcher provided complementary methodological expertise. The paper was co-authored based on an initial draft that was produced as part of the MSc dissertation.

### Data preprocessing and measurements

We scored the three questionnaires according to their guidelines. SC-IAT D-scores were computed using Greenwald’s revised algorithm (Greenwald et al., 2003), excluding 56 trials (<0.001% of total) with response times under 400 ms, but no participants due to rapid response rates (see *Supplementary Materials S1* for details).

Non-standardized responses (e.g. age, education, contact with Autistic people) were converted to numeric values. Age ranges were mapped linearly, and education levels were approximated as years of education. Gender was treated as a three-level factor (‘Male’, ‘Female’, ‘Other’). In our main analysis, political orientation was analysed as a scale after removing ‘Other’ (N = 3) and ‘Prefer not to say’ (N = 12) responses (though see section *Complementary analyses*).

Reading behaviour and trust ratings were numerically coded. We applied a quadratic transformation to reading frequency responses to better reflect actual exposure (details in *Supplementary Materials S2*). We derived two primary measures of reading behaviour:

1. *Overall exposure to newspapers*: This was the sum of reading frequency across all newspapers. It could range from no engagement at a score of 0 up to 160, corresponding to exposure to every 1 of the 10 newspapers on a daily basis.
2. *Selective engagement with specific newspapers (Reading preference for right-leaning tabloids)*. This measure was calculated as the difference between the reported reading frequency of four right-leaning tabloids (*Daily Express*, *Daily Mail*, *Daily Star* and *The Sun*) and three left-leaning broadsheets (*The Guardian*, *The Independent* and *The Observer*). The classification of newspapers by political orientation and reporting style was based on Karaminis et al. (2023; see also Balfour, 2019). Higher scores on this measure indicated more frequent reading of right-leaning tabloids relative to left-leaning broadsheets. Possible scores ranged from −48 (daily reading of all left-leaning broadsheets and none of the right-leaning tabloids) to 64 (daily reading of all right-leaning tabloids and none of the left-leaning broadsheets).

Similarly, we calculated two trust metrics: *Overall trust in newspapers* (mean trust rating across all newspapers, range: 0 to 40) and *Selective trust in specific newspapers* (difference in trust ratings between right-leaning tabloids and left-leaning broadsheets, range: −12 to 16).

### Main data analysis

#### Generalized additive models and weighting

Our main analysis employed regression models with explicit or implicit attitudes as outcome variables, and all other measures – demographics, contact with Autistic people, autism knowledge, the complementary attitude measure and newspaper reading/trust variables – as predictors. We used non-parametric generalized additive models (GAMs) via the *mgcv* package in R (Wood, 2017). GAMs were chosen for their flexibility in capturing non-linear effects without overfitting the data, and for not relying on strict parametric assumptions.

In our GAM models, we weighted the data by participants’ overall newspaper exposure, giving greater importance to those who read newspapers frequently. This approach addressed substantial variability in newspaper exposure, as nearly 20% of participants reported never reading newspapers and many others read them only infrequently (see *Supplementary Materials S3*). Weighting helped ensure that the relationship between reading preferences and attitudes towards autism was not unduly influenced by participants with minimal newspaper exposure.

#### Hierarchical partitioning

We also applied hierarchical partitioning to all GAMs using the *gam.hp* package in R (J. Lai et al., 2024) to determine the relative contribution of each predictor to the explained variance in outcome variables. This technique was necessary given that our models included multiple predictors with some strong associations (e.g. knowledge about autism and contact with Autistic people: *r_s_* = 0.37, p < 0.001; years of education and selective reading preference for right-leaning tabloids: *r_s_* = −0.31, p < 0.001; see *Supplementary Materials S3*). Such associations – known as collinearities (or concurvities in GAMs) – complicate interpretation, as they mean certain predictors may share explained variance or distort the effects of other predictors (suppressor effects) (for a discussion, see J. Lai et al., 2022a, 2022b, 2024).

Hierarchical partitioning addresses these challenges statistically while providing an intuitive way to interpret complex regression models. In particular, hierarchical partitioning involves quantifying each predictor’s unique contribution to explained variance based on the adjusted R² measure. Adjusted R² penalizes unnecessary model complexity by assigning negative values to predictors that do not improve explanatory power. In our analysis, such negative values were treated as zero contribution.

#### Post hoc analysis of knowledge about autism

Finally, since autism knowledge emerged as a key predictor of attitudes, we carried out a complementary analysis to assess how reading behaviour and trust contributed to variance in autism knowledge.

### Complementary data analyses

We also conducted a range of additional analyses to examine the extent to which the findings of the main analysis depended on specific decisions and assumptions. First, we assessed the impact of weighting the data by overall exposure to newspapers. To this end, we developed unweighted ‘baseline’ GAM models, in which overall newspaper exposure was one of the factors rather than being applied as a weight. In addition, as an alternative approach to weighting by exposure to newspapers, we excluded participants with minimal exposure to newspapers and then fitted unweighted GAMs to this reduced dataset.

Second, we examined the impact of excluding participants who reported their political orientation as ‘Other’ or ‘Prefer not to say’ (i.e. whose responses could not be positioned on the left–right political spectrum). We carried out a control analysis in which these responses were retained and political orientation was treated as an unordered categorical variable.

Finally, we assessed the impact of potential collinearity and concurvity between different predictors in our data. For this, we conducted an alternative analysis using model selection procedures, iteratively excluding predictors that exhibited high collinearity or concurvity. This resulted in a final GAM model with a reduced number of predictors.

The results of these additional analyses, along with comparisons to the main GAM models, are presented in the Supplementary Materials. To foreshadow some of the key findings, weighting the data by newspaper exposure substantially increased the explained variance in the outcome variables and uniquely revealed significant effects of variables related to newspaper reading behaviour. This was especially evident when comparing the main weighted models with the unweighted ‘baseline’ GAMs (*Supplementary Analysis S7 to S9*) which explained considerably less variance and yielded no significant effects for reading-behaviour-related variables. A similar but less pronounced pattern was observed when comparing the weighted models with those based on the reduced dataset (*Supplementary Analysis S13 to S15*), which accounted for less variance than the main weighted GAMs but more variance than the baseline models, and also captured some key effects of reading-behaviour-related variables.

The inclusion or exclusion of participants based on their political orientation did not alter the overall pattern of results (*Supplementary Analysis S10 to S12*). Similarly, the reduced predictor models (*Supplementary Analysis S16 to S18*) yielded results consistent with the main analysis, suggesting that potential collinearity or concurvity among predictors was not a major concern.

## Results

### Explicit attitudes

**Figure 2.**
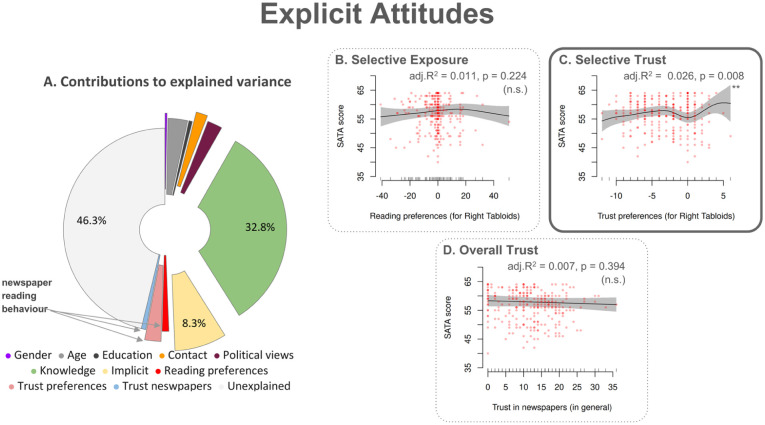
Explicit Attitudes – Factor contributions to explained variance and associations with newspaper reading behaviour variables. (a) The pie chart illustrates the contributions of various predictors to the variance that the GAM model for explicit attitudes explains. We assessed explicit attitudes using the Societal Attitudes Towards Autism (SATA) scale. We determined these contributions using hierarchical partitioning analysis for the GAM model with weighting by overall newspaper exposure. This procedure calculated the proportion of adjusted R² corresponding to individual predictors. The pie chart shows segments corresponding to different factors in the same order as the legend (starting at the top and moving clockwise). The distance of a segment from the centre indicates the significance of the corresponding factor (greater distance signifies lower p-values). The light grey segment represents variance that the GAM model left unexplained. (b–d) These panels depict the partial effects of three predictors related to newspaper reading preferences on attitudes towards autism, based on estimates from the GAM model. Panel (b) shows the effect of preferences for right-leaning tabloids over left-leaning broadsheets, Panel (c) illustrates the effect of trust preferences for right-leaning tabloids, and Panel (d) presents the effect of overall trust in newspapers. The framing of each panel denotes the significance of the partial effects: a dotted line indicates a non-significant effect, a solid line signifies a significant effect and a bold solid-line frame highlights a highly significant effect. The text at the top of each subplot shows the individual contribution to adjusted R^2^ and the corresponding p-values (*p < 0.05; **p < 0.01; ***p < 0.001). As an example, Panel (c) demonstrates a non-linear trend where SATA-scores tend to increase when selective trust to right-leaning tabloids increases, suggesting that participants with higher levels of trust specifically in these newspapers tend to present relatively favourable explicit attitudes towards autism. This effect, which contributes 2.6% of the explained variance, is moderately significant.

As shown in Figure 2(a) (see also *Supplementary Materials S4*), the GAM model weighted by overall newspaper exposure explained 53.75% of the variance in explicit attitudes (mean SATA score = 56.38, *SD* = 5.33; *Supplementary Table S1*). This was 17.9 percentage points higher than in the unweighted baseline model (*Supplementary Materials S7*).

Autism knowledge accounted for the largest share of explained variance (individual contribution to adjusted R² = 32.83%) through a significant non-linear effect (p < 0.001), whereby greater knowledge was generally linked to more positive explicit attitudes. Implicit attitudes contributed another 8.28% of explained variance (p < 0.001), through a non-linear effect. More specifically, explicit attitudes tended to improve with more positive implicit attitudes; however, there was a slight decline among participants with the most positive implicit attitudes.

#### Reading-behaviour variables

Newspaper reading behaviour collectively explained 4.40% of the variance in explicit attitudes – an increase of 4.03 percentage points compared to the unweighted model (*Supplementary Materials S7*). Reading preferences for right-leaning tabloids (Figure 2(c)) accounted for 1.10% of the variance but were not a significant predictor (p = 0.224). In contrast, trust preferences (Figure 2(d)) significantly predicted explicit attitudes (p = 0.008), contributing 2.58% of the variance. A non-linear effect indicated that greater trust in right-leaning tabloids tended to be associated with slightly more positive explicit attitudes. Overall trust in newspapers (Figure 2(e)) explained 0.72% of the variance but was not a significant predictor (p = 0.39).

#### Other factors

Several predictors unrelated to newspaper reading also contributed significantly to the weighted GAM model, though each explained relatively little variance. Age accounted for 3.10%, following an inverted-U pattern (p = 0.035), whereby middle-aged participants had the most positive explicit attitudes. Political views explained 2.39% of the variance in explicit attitudes and, unexpectedly, left-leaning participants tended to report less positive explicit attitudes than right-leaning ones (p = 0.001). Contact with Autistic people (1.87%) showed a linear effect (p < 0.001), and again unexpectedly, closer contact was linked to slightly less positive explicit attitudes. Gender accounted for 0.32% of the variance, with males reporting more favourable attitudes than females (p = 0.003). Years of education contributed 0.56%, with higher attainment associated with slightly less positive explicit attitudes (p = 0.011).

### Implicit attitudes

**Figure 3.**
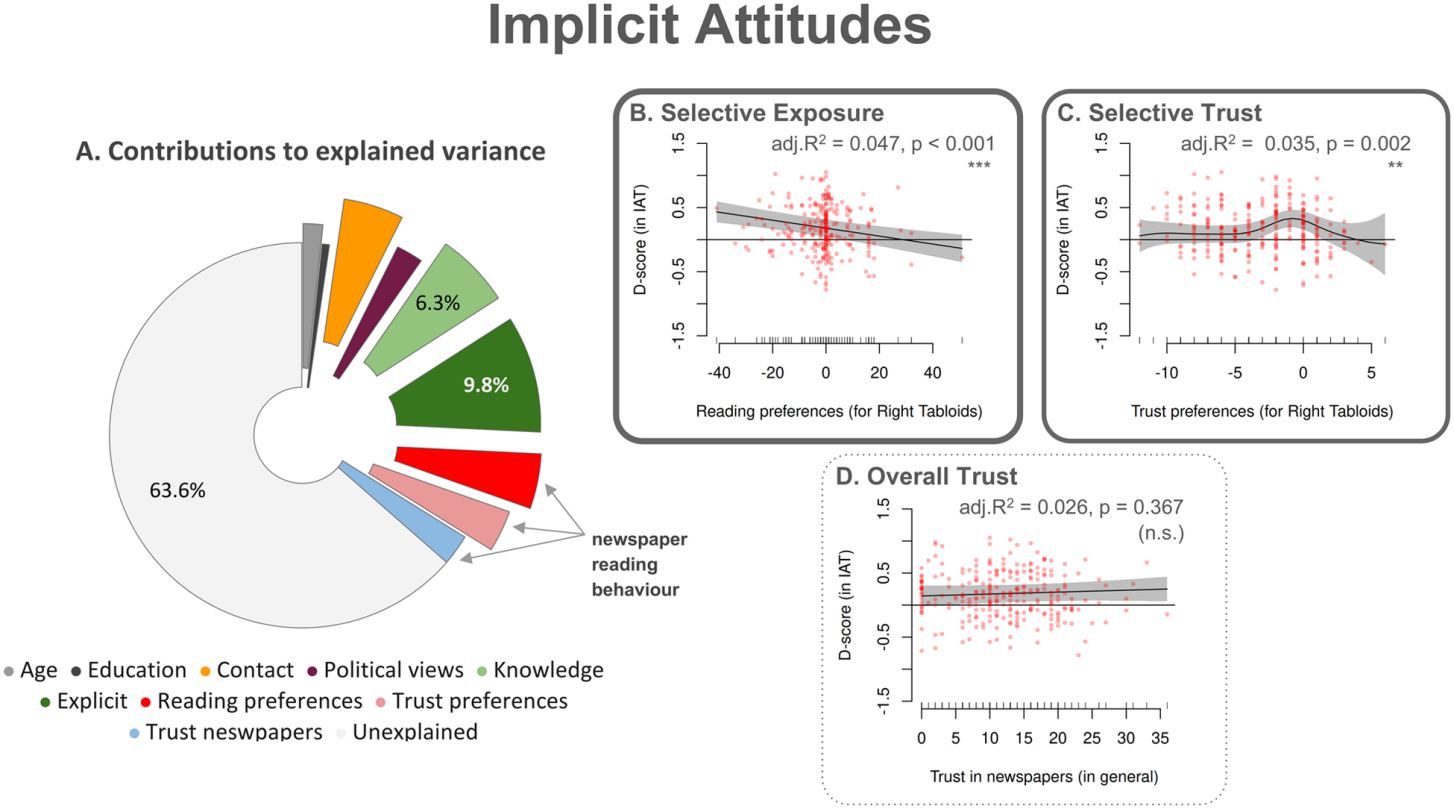
Implicit attitudes – Factor contributions to explained variance and associations with newspaper reading behaviour variables. (a) The pie chart illustrates the contribution of various predictors to the variance that the GAM model for implicit attitudes explains. We assessed implicit attitudes towards autism using a Single Category Implicit Association Test (SC-IAT). (b–d) Panels depict the partial effects of three predictors relevant to newspaper reading preferences on implicit attitudes. The configuration of this figure is identical to Figure 1; the reader may refer to its caption for further details.

Figure 3(a) (and *Supplementary Materials S5*) shows results from the weighted GAM model for implicit attitudes. This model explained 36.39% of the variance (mean D-score = 0.157, SD = 0.15; *Supplementary Table S1*), a 20.09 percentage-point increase over the unweighted baseline model (see *Supplementary Materials S8*).

The largest contributor to explained variance in implicit attitudes was explicit attitudes, accounting for 9.86% via a highly significant non-linear effect (p = 0.007), whereby more positive explicit attitudes were generally linked to more positive implicit attitudes. Knowledge about autism explained 6.36% of the variance, via a non-linear pattern (p < 0.001), whereby, unexpectedly, higher AAS scores were associated with slightly less favourable implicit attitudes.

#### Reading-behaviour variables

Reading behaviour variables collectively accounted for 10.82% of the variance in implicit attitudes, an 8.22 percentage-point increase over the unweighted baseline model (see *Supplementary Materials S8*). Reading preferences for right-leaning tabloids (Figure 3(b)) accounted for 4.67% via a highly significant linear effect (p < 0.001), whereby greater exposure was clearly linked to less favourable implicit attitudes. Trust preferences (Figure 3(c)) explained 3.51% through a significant non-linear effect (p = 0.002). Specifically, those with neutral trust showed the most favourable implicit attitudes, while participants with the highest trust in right-leaning tabloids appeared to present the least favourable implicit attitudes. Overall trust in newspapers (Figure 3(e)) explained 2.64% of the variance in implicit attitudes but was not a significant predictor (p = 0.367).

#### Other predictors

Among other predictors, contact accounted for 4.77% of the variance in implicit attitudes, with people with more contact with Autistic people having more positive implicit attitudes (p < 0.001). Political views explained 2.25%, with people reporting more left-leaning views having more positive implicit attitudes (p = 0.008).

### Knowledge about autism

**Figure 4.**
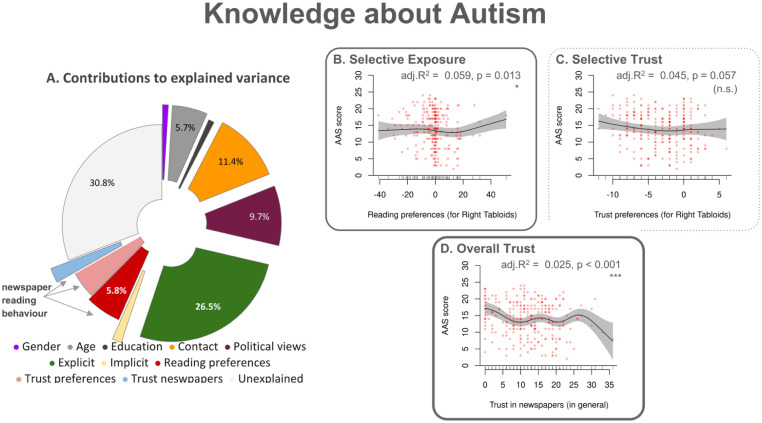
Knowledge about Autism – Factor contributions to explained variance and associations with newspaper reading behaviour variables. (a) The pie chart shows the contribution of various predictors to the variance that the GAM model for knowledge about autism explains. We assessed knowledge about autism using the Autism Awareness Scale (AAS). (b–d) Panels illustrate the partial effects of three predictors relevant to newspaper reading preferences on knowledge about autism. The configuration of this figure is identical to Figure 1; the reader may refer to its caption for further details.

In the post hoc analysis of predictors of autism knowledge (Figure 4; *Supplementary Materials S6*), the weighted GAM model explained 70.25% of the variance – 23.63% more than the unweighted model (*Supplementary Materials S9*). Explicit attitudes were the largest contributor (26.58%; p < 0.001), with better attitudes clearly linearly linked to greater knowledge. Closer contact with Autistic people also predicted greater knowledge in a non-linear pattern (11.47%; p < 0.001). Political views explained 9.80% of the variance in AAS scores, with more left-leaning participants having more accurate knowledge (p < 0.001). Age accounted for 5.83%, with older ages linked to less accurate knowledge about autism (p < 0.001). Implicit attitudes made a smaller contribution of 1.63% to explained variance via a non-linear effect (p < 0.001), whereby some participants with negative implicit attitudes featured slightly higher autism knowledge. Finally, gender contributed 1.04% of explained variance, with males showing less accurate knowledge than females (p = 0.037).

#### Reading-behaviour variables

Reading behaviour variables collectively explained 12.84% of the variance in autism knowledge, up by 9.53 percentage points compared to the unweighted model (*Supplementary Materials S9*). Reading preferences (Figure 4(b)) contributed 5.88% via a significant non-linear effect (p = 0.013), with some participants with strong preferences towards right-leaning tabloids showing sharp increases in autism knowledge. Trust in right-leaning tabloids (Figure 4(c)) explained 4.56%, but this effect was a non-significant trend (p = 0.057). In contrast, overall trust in newspapers explained 2.50% through a significant non-linear effect (p < 0.001), with higher overall trust associated with less accurate autism knowledge.

## Discussion

This cross-sectional study explored the relationship between newspaper reading preferences and public attitudes towards autism. We examined how implicit and explicit attitudes relate to detailed newspaper reading habits, including self-reported reading frequency and trustworthiness ratings for individual newspapers. In addition, we considered a range of other variables previously associated with attitudes towards autism.

Crucially, a reading preference for right-leaning tabloid newspapers emerged as a reliable predictor of more negative implicit attitudes. While caution is warranted in drawing causal conclusions from cross-sectional data, this finding suggests that exposure to newspaper content may play a role in the dynamic process of attitude formation towards autism. Our combined results are also consistent with key tenets of cultivation theory, which posits that media effects accumulate over time through repeated exposure (Arendt, 2010; Gerbner et al., 2002; Happer & Philo, 2013; Jamieson & Waldman, 2003). Indeed, in our data, reading habits was a reliable predictor of attitudes only when overall newspaper exposure was accounted for, as revealed by the comparison of weighted and unweighted GAM analyses.

In contrast, a preference for right-leaning tabloids showed limited associations with explicit attitudes. Although causal interpretations should again be approached with caution, this discrepancy in the relationship between reading preferences and the two attitude measures aligns with theoretical models proposing that media-driven stereotypes primarily influence implicit attitudes, which may later inform explicit beliefs (Arendt & Northup, 2015). From this perspective, implicit attitudes correspond to an earlier stage of the attitude formation process and may therefore be more directly influenced by reading habits compared to explicit attitudes (though see Holroyd et al., 2017 for a critique of philosophical and empirical issues pertinent to implicit attitudes).

Unlike reading frequency, trust in right-leaning tabloids was associated with both explicit and implicit attitudes towards autism, though in more complex and non-linear ways. Notably, some participants who reported higher trust in these outlets expressed relatively favourable explicit attitudes, yet simultaneously exhibited more negative implicit attitudes. This difference might stem from social desirability influencing self-reported measures, while implicit attitudes, being more automatic and less consciously controlled, remain relatively unchanged (Dickter et al., 2020; Dovidio et al., 1997; D. R. Jones et al., 2021).

The fact that this dissonance was especially pronounced among individuals who trusted right-leaning tabloids may suggest that these individuals are particularly susceptible to social desirability bias. However, it is difficult to determine the underlying causes of this effect. One possible explanation is that right-leaning tabloids frequently frame autism through sensationalistic narratives that emphasize individual responsibility and blame frames, which may subtly reinforce stigma (Karaminis et al., 2025; Molek-Kozakowska, 2013). Individuals who trust these sources may be more likely to internalize such framings at a less accessible level, even as they consciously attempt to align their explicit attitudes with socially desirable norms – thereby creating a dissonance between implicit and explicit responses (see also Arendt & Northup’s (2015) findings on media framing of crime, as well as Zappettini (2021) on tabloid coverage of Brexit).

However, crucially, an alternative possible explanation of our findings is that the trust measure does not solely capture attitudes towards individual newspapers, but reflects a broader constellation of pre-existing political beliefs and personality traits. One relevant factor is the acceptance of inequality, which has shown to predict attitudes towards autism (Cheng et al., 2025; Gillespie-Lynch et al., 2021). For example, Cheng et al. (2025) found that constructs such as vertical individualism (an orientation towards hierarchy and competition) and horizontal collectivism (an orientation towards equality and group cohesion) predict both explicit and implicit attitudes towards autism in cross-cultural contexts. Arguably, these broader ideological and psychological dispositions could influence not only an individual’s attitudes towards Autistic people, but also attitudes towards some other disability groups or marginalized groups. Furthermore, such ideological and psychological dispositions might influence how much individuals trust and how likely they are to engage with content from right-leaning media.

The extent to which these broader factors drive the patterns observed in our data warrants further investigation. On the one hand, it is plausible that reading behaviour and trust are shaped by deeper psychological and social orientations – factors which may not be adequately captured by simple self-report measures of political ideology (see, for example, Westfall & Yarkoni, 2016 about challenges in measuring and accounting for confounding factors). On the other hand, the effects of both trust and reading preferences in our data appear to be moderated by overall newspaper exposure. This suggests some degree of specificity in the effects of newspaper reading behaviour on attitudes towards autism, rather than that these patterns should be primarily attributed to general ideological orientation (which underlies both autism attitudes and newspaper preferences). Nevertheless, this study did not consider the extent to which participants actually engage with newspaper content about autism, nor the degree to which attitudes towards autism overlap with attitudes towards other forms of neurodivergence, disability or marginalized groups.

Finally, a post hoc analysis of autism knowledge suggested that greater trust in newspapers was associated with less accurate knowledge about autism. This may suggest that a more critical or discerning approach to media consumption is important for fostering accurate understanding of autism.

### Implications for the inclusion and acceptance of Autistic People

This study focused on newspaper reading behaviours, particularly selective engagement with and trust in right-leaning tabloids as examples of outlets that mention autism less frequently but tend to present more stereotypical and stigmatizing portrayals compared to left-leaning broadsheets (Karaminis et al., 2023). However, it is important to acknowledge that deficit-focused or stigmatizing representations of autism are not exclusive to these outlets. A large body of research indicates that such portrayals are widespread across the media landscape, encompassing various newspaper types as well as other platforms (Akhtar et al., 2022; Baroutsis et al., 2023; Jawed et al., 2023; S. C. Jones et al., 2023; Karaminis et al., 2023; Mittmann et al., 2024). Supporting this view, a recent participatory study found that Autistic experts perceived only minor differences in autism-related coverage across different newspaper types (Karaminis et al., 2024).

Taken together, our findings suggest that the quality of the representation of autism in newspapers is an important factor involved in the dynamics of construction of implicit and explicit attitudes towards autism. However, crucially, these implications are not limited to print journalism; they likely extend to other forms of media consumption – including television, film, digital platforms and social media – which often reach broader and more diverse audiences and may exert even greater influence on public perceptions. In this wider context, media outlets can contribute to either a ‘virtuous circle’, where respectful and informed coverage promotes more favourable attitudes towards Autistic people, or a ‘vicious circle’, where sensationalistic, stereotypical representations reinforce stigma (O’Connor & Downey, 2024). Editors, journalists and media professionals can foster more positive attitudes by producing content that reflects the lived experiences of Autistic people and promotes nuanced, strengths-based perspectives. Autistic advocates, community leaders and media professionals – including Autistic journalists – are especially well-positioned to drive these changes. Their involvement can guide news outlets towards more empathic, inclusive reporting, ultimately advancing societal acceptance.

Readers (and listeners and viewers) may also play a vital role by approaching autism coverage with a critical eye, that is, questioning sensational headlines, verifying information through multiple sources and seeking out first-person accounts from Autistic individuals. Moreover, readers, listeners and viewers can influence future reporting by providing feedback – such as highlighting biased language or praising balanced coverage – to encourage editors and journalists to refine their practices. Over time, consistent engagement may help foster more informed, respectful discussions of autism.

### Strengths, limitations and future work

To our knowledge, this study is the first to examine how newspaper reading behaviour relates to attitudes towards autism, while also accounting for a range of other variables known to influence both attitudes and reading preferences. Our findings extend previous research on press representations of autism – and media portrayals more broadly – by providing empirical evidence that bridges this area with work on implicit and explicit attitudes. Furthermore, our statistical approach provides a robust framework that can be readily applied to evaluate and quantify the role of other forms of media in the dynamics of attitude formation towards autism, as well as towards other disabilities and marginalized groups.

Despite these contributions, several limitations should be acknowledged. Most notably, our survey-based approach identified associations between key factors and attitudes, but does not permit causal inference. Understanding the mechanisms underlying these relationships will require longitudinal studies or experimental designs that manipulate media exposure to observe changes in attitudes over time.

Our statistical modelling methods explained more than half of the variance in explicit attitudes and one-third of the variance in implicit attitudes, and quantified the contribution of variables related to newspaper preferences in explaining this variance. However, a substantial proportion of variance remains unexplained, highlighting the complexity of human attitudes and particularly implicit ones, which tend to be more deeply ingrained, less consciously accessible and more resistant to change (Dickter et al., 2020; Dovidio et al., 1997, 2002; Gillespie-Lynch et al., 2015, 2021; Wilson et al., 2000). Importantly, this study did not account for several intrinsic and environmental factors that may shape both attitudes and media engagement. For example, beyond ideological orientations such as acceptance of inequality, socioeconomic status may influence both attitudes and newspaper preferences.

In addition, the role of broader media ecosystems – including social media – warrants further investigation. According to the 2024 Reuters Institute Digital News Report, only 34% of people in the United Kingdom now consume newspapers, either in print or online, and this figure has declined in recent years (Newman et al., 2024). Future research should adopt a more nuanced approach that captures engagement across both traditional and digital platforms, particularly within algorithm-driven environments that may reinforce echo chambers and limit exposure to alternative viewpoints (Flaxman et al., 2016).

The sampling strategy also introduces limitations. Participants were recruited via an online platform, which may attract individuals with specific characteristics (Peer et al., 2021). Compared to the general population, our sample included a higher proportion of women, younger adults and individuals with higher educational attainment and left-leaning political views, which may have introduced sampling biases. Unexpectedly, men in our sample reported more favourable explicit attitudes than women, and participants with higher educational attainment or greater contact experience reported less favourable explicit attitudes, contrary to earlier findings (Kim et al., 2023b). Such patterns may reflect idiosyncrasies within our sample. Individuals engaging with online crowdsourcing platforms can be regarded as a ‘professional community’, survey-literate and often highly familiar with research methods (i.e. non-naïve) (Chandler et al., 2019; Peer et al., 2017). These participants may also develop unusual familiarity with some recurring research themes, including autism – though often approached from a medicalized perspective (Botha & Cage, 2022). Their individual characteristics of these experienced participants may shape how they respond to questionnaires, particularly in relation to sensitivity to social desirability norms. For example, some men may moderate the expression of relatively negative attitudes, whereas participants with extensive contact experience or higher education may feel a stronger sense of authority that enables them to voice more critical perspectives. These interpretations remain speculative, and replication with more diverse samples is needed to better capture real-world variability in both experiences and attitude.

In addition, future work should consider unmeasured influences, such as the quality (rather than merely the quantity) of contact with Autistic people. It may also be important to consider whether participants’ educational backgrounds relate specifically to healthcare or human-centred professions, rather than relying on general educational attainment (Gardiner & Iarocci, 2014; McManus et al., 2011). More nuanced assessments of autism-related knowledge and attitudes in everyday contexts – particularly those developed in genuine collaboration with Autistic people – would also be valuable and directly relevant to the Autistic experience (S. C. Jones, 2022). Another methodological issue to be addressed concerns the order in which questions are asked; for example, in our study, asking participants to report their degree of contact with Autistic people at the beginning of the survey may have primed their subsequent responses to the attitude measures by drawing attention to their specific experiences with Autistic people.

Finally, it is important to examine how attitudes vary across subgroups within the Autistic community, especially those who are often less visible or more frequently misrepresented in the media, such as Autistic adults (O’Connor & Downey, 2024), Autistic women and LGBTQIA+ Autistic people (Karaminis et al., 2025). Promoting understanding and acceptance of these intersectional identities is a particularly urgent priority (Botha & Gillespie-Lynch, 2022).

## Supplemental Material

sj-pdf-1-aut-10.1177_13623613251394523 – Supplemental material for The relationship between newspaper reading preferences and attitudes towards autism Supplemental material, sj-pdf-1-aut-10.1177_13623613251394523 for The relationship between newspaper reading preferences and attitudes towards autism by Marta Dickinson and Themis Karaminis in Autism
