# Supplementary material for: The relationship between newspaper reading preferences and attitudes towards autism
Source: Autism. 2025 Dec 3;30(3):574–91. doi: 10.1177/13623613251394523 (PMC12923640; doi:10.1177/13623613251394523)
Supplement: sj-pdf-1-aut-10.1177_13623613251394523 – Supplemental material for The relationship between newspaper reading preferences and attitudes towards autism [file sj-pdf-1-aut-10.1177_13623613251394523.pdf]

Supplementary Materials

The Relationship Between Newspaper Reading Preferences and Attitudes Toward Autism.

S1. Further information on the Single Category Implicit Association Test (SC-IAT)

Rationale/Relevance to the main paper

This section provides additional details on the scoring procedures for the SC-IAT.

SC-IAT Scoring

We scored the SC-IAT using the *cleanIAT* function from the *iatgen* R package (Carpenter et al., 2019), which implements the revised scoring algorithm proposed by Greenwald et al. (2003). This algorithm removes trials with response latencies greater than 10,000 ms or less than 400 ms. There were 56 such trials in our data corresponding to 0.001%. Additionally, the algorithm identifies participants whose response latencies are less than 300 ms on more than 10% of trials for exclusion. However, no participants met this exclusion criterion in our data.

We calculated D-scores by subtracting participants' mean response latencies for the "negative" blocks from their mean response latencies for the "positive" blocks. Following the D600 procedure outlined in Greenwald et al. (2003, p. 214, right column),[2] we scored errors in the SC-IAT by adding a penalty of 2 standard deviations (SD) of the correct-response latencies to the block mean of correct trials.

S2. Measuring reading behavior

Rationale/Relevance to the main paper

This section offers further details on measures of reading behavior.

Reading behavior measures

We mapped the responses for reading preferences for each newspaper quadratically to numbers between 0 and 25 to better reflect actual reading frequency. The mapping was as follows: "Never" → 0, "A few times a year" → 2, "A few times a month" → 9, "A few times a week" → 19, "Daily" → 25. We selected this transformation to better approximate the frequency of exposure to each newspaper. For trustworthiness ratings, we mapped responses linearly to numbers between 0 and 4 as follows: "Not trustworthy" → 0, "Very trustworthy" → 4.

Using these transformed values, we computed the following measures:

- **Overall Exposure to Newspapers:** This measure represents the summed reading frequency across all newspapers. It ranges from 0 (no engagement) to 160, corresponding to daily exposure to all 10 newspapers.
- **Selective Exposure to Newspapers (Reading Preference for Right-Leaning Tabloids)**: This measure is the difference between the reading frequency of the four right-leaning tabloids (Daily Express, Daily Mail, Daily Star, and The Sun) and the reading frequency of the three left-leaning broadsheets (The Guardian, The Independent, and The Observer). Positive values indicate a greater reading preference for right-leaning tabloids, while negative values indicate a greater preference for left-leaning broadsheets. The measure ranges from −48 (daily exposure to all left-leaning broadsheets and no exposure to right-leaning tabloids) to 64 (daily exposure to all right-leaning tabloids and no exposure to left-leaning broadsheets).
- **Overall Trust in Newspapers:** This is the mean trust rating across all newspapers, with a potential range of 0 (all newspapers judged as not trustworthy) to 40 (all newspapers judged as very trustworthy).

- **Selective Trust in Newspapers (More Trust in Right-Leaning Tabloids):** This measure is the difference between trust ratings of right-leaning tabloids and left-leaning broadsheets. It ranges from −12 (lowest trust in right-leaning tabloids and highest trust in left-leaning broadsheets) to 16 (highest trust in right-leaning tabloids and lowest trust in left-leaning broadsheets).

Supplementary Figure S1 presents histograms showing the distribution of the four reading behavior measures, while **Supplementary Table S2** provides descriptive statistics (means, medians, standard deviations, and ranges) for these measures.

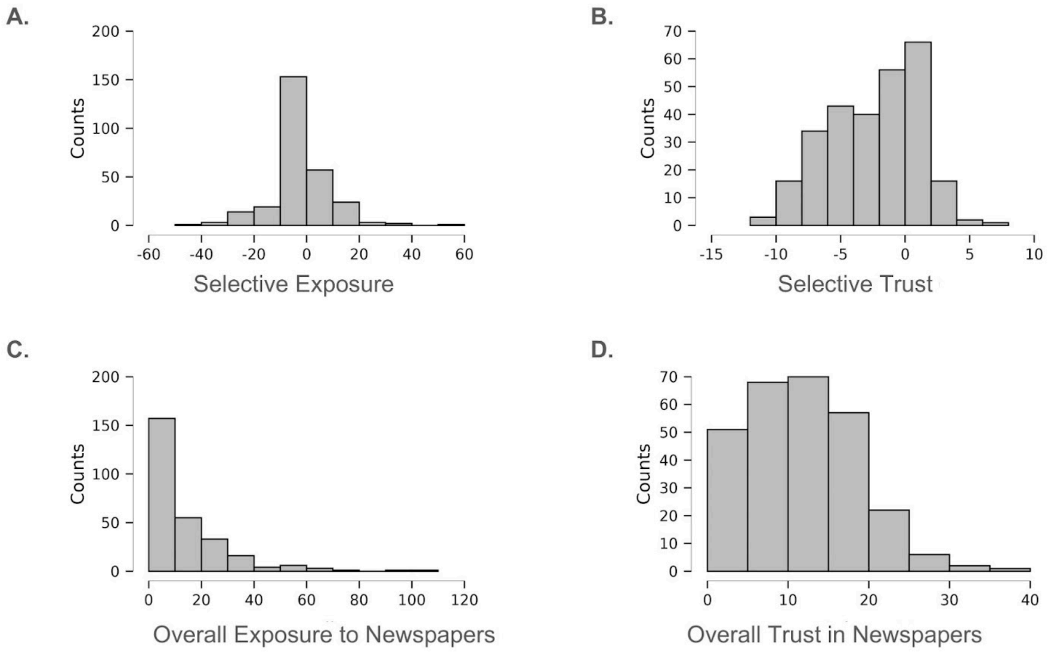

Supplementary Figure S1. Histograms for the distribution of the four measures of reading behavior. **A. Selective Exposure to Newspapers:** Reading preference for right-leaning tabloids versus left-leaning broadsheets. **B. Selective Trust in Newspapers:** Higher trust in right-leaning tabloids compared to left-leaning broadsheets. **C. Overall Exposure to Newspapers:** Total reading frequency across all newspapers. **D. Overall Trust in Newspapers:** Sum of trust ratings across all newspapers.

S3. Descriptive statistics and correlations

Rationale/Relevance to the main paper

This section provides results from a preliminary analysis of the data, which examined descriptive statistics, distributional characteristics, and rank-order correlations between different measures.

Descriptive Statistics

Supplementary Table S1 summarises descriptive statistics.

Supplementary Table S1. Descriptive statistics for attitudes toward autism, knowledge about autism, and reading behavior measures.

Variables	Mean	Median	Standard Deviation	Min	Max	Normality check p-value
SATA (Explicit)	56.38	57.00	5.33	40	64	p < 0.001
D-score (Implicit)	0.16	0.15	0.35	−0.782	1.05	p = 0.65
AAS (Knowledge)	13.88	14	5.36	−1	24	p < 0.001
Overall exposure to newspapers	13.01	7	16.10	0	106	p < 0.001
Overall trust in newspapers	11.96	12	7.30	0	36	p < 0.001
Selective exposure	−0.88	0	10.84	−41	51	p < 0.001
Selective trust	−3.53	-2	3.53	−12	6	p < 0.001

**Note*: SATA: Societal Attitudes Toward Autism (Flood et al, 2013); AAS: Autism Awareness Scale (Gillespie et al., 2015; Tipton et al., 2014).

Rank-Order correlations

Supplementary Table S2. Spearman's Rank Correlation Matrix

Variable		1	2	3	4	5	6	7	8	9	10	11
**1. AGE**	*Sp. rho*	—										
	p-value	—										
**2. EDUCATION (YEARS)**	*Sp. rho*	**-0.160**	—									
	p-value	**0.008 ****	—									
**3. POLITICAL VIEWS (responses on the Left- to Right-leaning scale)**	*Sp. rho*	**-0.221**	**0.125**	—								
	p-value	**< .001*****	**0.044***	—								

Variable		1	2	3	4	5	6	7	8	9	10	11
**4. CONTACT**	*Sp. rho*	**-0.123**	0.095	0.046	—							
	p-value	**0.041***	0.116	0.455	—							
**5. KNOWLEDGE ABOUT AUTISM**	*Sp. rho*	**-0.239**	**0.119**	**0.332**	**0.374**	—						
	p-value	**< .001*****	**0.049***	**< .001*****	**< .001*****	—						
**6. EXPLICIT ATTITUDES**	*Sp. rho*	**-0.227**	0.018	**0.237**	**0.190**	**0.592**	—					
	p-value	**< .001*****	0.770	**< .001*****	**0.001*****	**< .001*****	—					
**7. IMPLICIT ATTITUDES**	*Sp. rho*	-0.116	0.051	0.113	**0.218**	**0.132**	**0.212**	—				
	p-value	0.053.	0.399	0.068.	**< .001*****	**0.028***	**< .001*****	—				

Variable		1	2	3	4	5	6	7	8	9	10	11
**8. SELECTIVE READING (Preference for Right-Leaning Tabloids)**	*Sp. rho*	0.013	**-0.305**	**-0.316**	0.039	**-0.127**	-0.085	-0.061	—			
	p-value	0.834	**< .001*** **	**< .001*** **	0.515	**0.035***	0.159	0.310	—			
**9. SELECTIVE TRUST (Trust in Right-Leaning Tabloids)**	*Sp. rho*	-0.022	**-0.314**	**-0.293**	-0.004	**-0.132**	-0.048	-0.084	**0.586**	—		
	p-value	0.715	**< .001*** **	**< .001*** **	0.948	**0.028***	0.422	0.163	**< .001*** **	—		
**10. OVERALL EXPOSURE TO NEWSPAPERS**	*Sp. rho*	**0.139**	**0.153**	-0.012	-0.042	-0.018	-0.033	0.002	**-0.171**	**-0.203**	—	
	p-value	**0.020***	**0.011***	0.842	0.490	0.769	0.581	0.975	**0.004** **	**< .001*** **	—	
**11. OVERALL TRUST IN NEWSPAPERS**	*Sp. rho*	**0.182**	-0.088	**-0.197**	-0.060	**-0.130**	**-0.143**	-0.043	**0.134**	-0.055	**0.309**	—
	p-value	**0.002** **	0.145	**0.001** **	0.319	**0.030***	**0.017***	0.481	**0.026***	0.363	**< .001*** **	—

Note. Sp. rho: Spearman's Rho. The number of observations (N) is 277 for all pairwise associations, except for those involving POLITICAL VIEWS, where N = 266 (see cells with blue ink). This is as the calculation of these correlations did not include participants who responded "Other" or "Prefer not to say" to the question about political views. *: $p < .05$, **: $p < .01$, ***: $p < .001$

S4. Further details on the weighted GAM model for Explicit attitudes

Rationale/Relevance to the main paper

This section presents detailed results for the parametric coefficients, which correspond to categorical predictors in the GAM model for **explicit attitudes**, namely gender and political views, as well as results for the "smooth" terms corresponding to the non-linear predictors in the same GAM model Additionally, **Supplementary Figure S2** illustrates the partial effects of significant terms not related to reading behavior (for the partial effects of reading preference variables see **Figure 1** in the main paper).

Categorical Predictors

Supplementary Table S3. Estimates for Parametric coefficients (weighted GAM model for Explicit attitudes).

	**Estimate**	**Std. Error**	**t value**	**p-value**
GENDERMale	1.5859	0.5231	3.032	0.0027 **
GENDEROther	1.9337	5.5133	0.351	0.7261

Note. *: p < .05, **: p < .01, ***: p < .001, Std. Error: Standard Error

Non-Linear Predictors

Supplementary Table S4. Estimates for Smooth terms (weighted GAM model for Explicit attitudes).

Term	edf	Ref.edf	F	p-value
s(AGE)	2.345	2.709	3.476	0.03533 *
s(EDUCATION_YEARS)	1.000	1.001	6.582	0.01092 *
s(CONTACT)	1.000	1.001	17.025	5.18e-05 ***
s(POLITICAL)	2.737	2.933	5.767	0.00128 **
s(KNOWLEDGE)	4.700	5.839	31.594	< 2e-16 ***
s(IMPLICIT)	3.665	4.534	11.970	< 2e-16 ***
s(Read.Right.Tabloids)	2.687	3.412	1.456	0.22361
s(Trust.Right.Tabloids)	5.788	7.058	2.860	0.00796 **
s(Trust.Newspapers.Amount)	1.000	1.000	0.728	0.39446

Note. *: $p < .05$, **: $p < .01$, ***: $p < .001$, edf (Estimated Degrees of Freedom): Represents the complexity of the smooth term. A value of 1 indicates an effectively linear effect, while higher values suggest increasing non-linearity. Ref. edf (Reference Degrees of Freedom): The maximum potential degrees of freedom that the smooth term could use, based on the basis dimension specified for the term in the model.

Effects of Other Factors (not related to reading behavior)

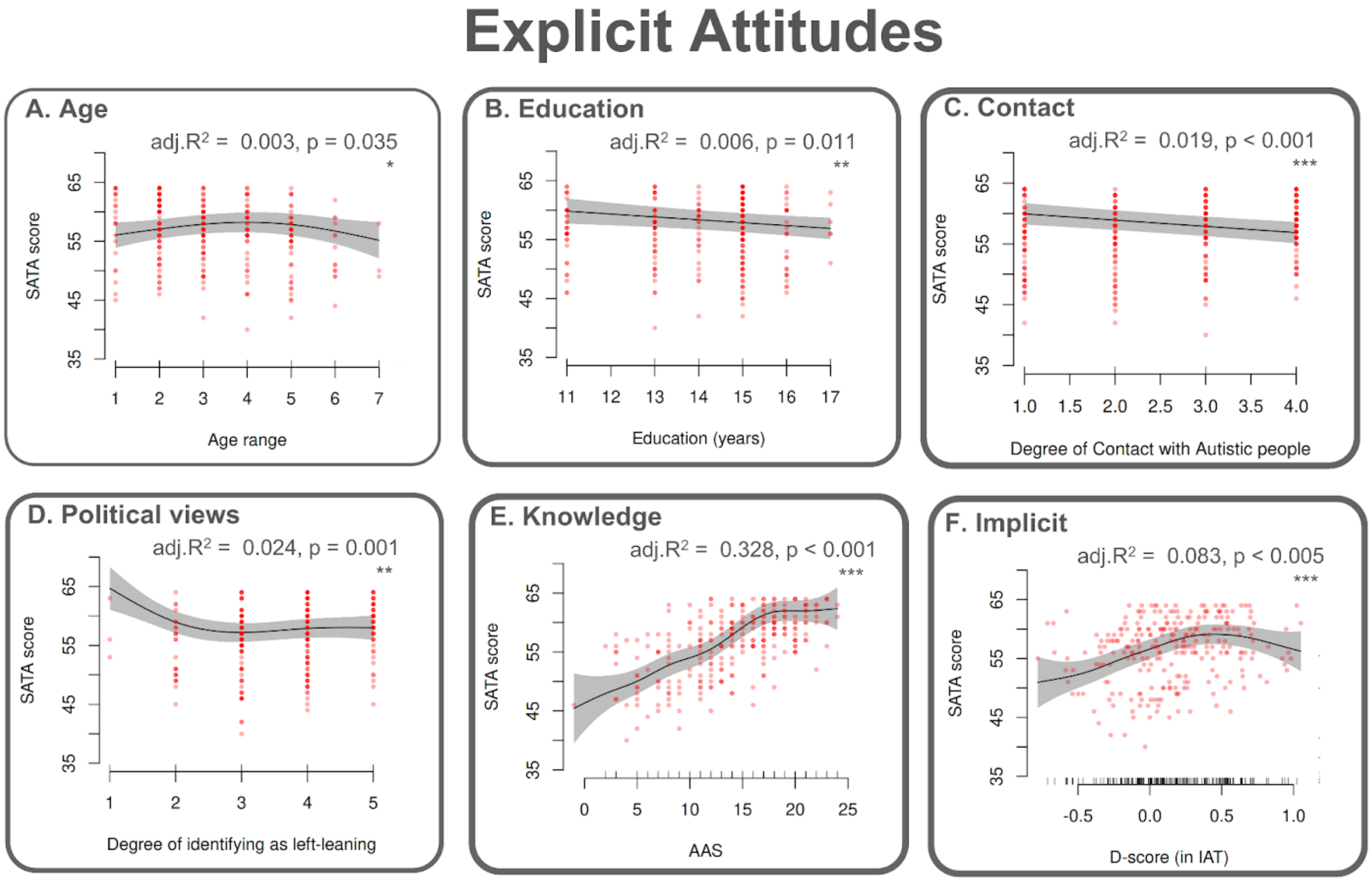

Supplementary Figure S2.

This figure illustrates the partial effects of five predictors of **explicit attitudes toward autism**, as measured with the SATA scale. These predictors, which are unrelated to newspaper reading preferences, are significant in the GAM model. **Panel A** shows the effect of age, **Panel B** presents the effect of educational attainment, **Panel C** illustrates the effect of prior contact with Autistic people, **Panel D** presents the effect of self-reported political leaning, **Panel E** depicts the effect of knowledge about autism (measured with the AAS), and **Panel F** shows the effect of implicit attitudes (estimated with the D-score measure from the SC-IAT). The framing of each panel indicates the significance of the partial effects: a thin solid line signifies a significant effect, while a bold solid-line frame highlights a highly significant effect. The text at the top of the plot shows the individual contribution to the adjusted R^2 and the corresponding p-values (*: $p < .05$, **: $p < .01$, ***: $p < .001$).

S5. Further details on the weighted GAM model for Implicit attitudes

Rationale/Relevance to the main paper

This section provides detailed results for the parametric coefficients, representing the categorical predictors in the GAM model for **implicit attitudes**, specifically gender and political views, along with the results for the "smooth" terms, which correspond to the non-linear predictors in the same GAM model. Additionally, **Supplementary Figure S3** illustrates the partial effects of significant terms unrelated to reading preferences (the partial effects of reading preference variables are presented in **Figure 3** of the main paper).

Categorical Predictors

Supplementary Table S5. Estimates for Parametric coefficients (weighted GAM model for Implicit attitudes).

	**Estimate**	**Std. Error**	**t value**	**p-value**
GENDERMale	-0.08641	0.04120	-2.098	0.037 *
GENDEROther	-0.19071	0.41991	-0.454	0.650

Note. *: p < .05, **: p < .01, ***: p < .001, Std. Error: Standard Error

Non-Linear Predictors

Supplementary Table S6. *Estimates for Smooth terms (weighted GAM model for Implicit attitudes).*

Term	edf	Ref.edf	F	p-value
s(AGE)	2.479	2.799	4.052	0.021105 *
s(EDUCATION_YEARS)	1.644	1.989	0.951	0.360576
s(CONTACT)	1.379	1.637	8.772	0.000497 ***
s(POLITICAL)	2.201	2.546	4.159	0.007809 **
s(KNOWLEDGE)	5.370	6.192	4.316	0.000311 ***
s(EXPLICIT)	5.446	6.647	6.719	1.06e-06 ***
s(Read.Right.Tabloids)	1.000	1.000	18.336	2.74e-05 ***
s(Trust.Right.Tabloids)	5.341	6.577	3.451	0.001858 **
s(Trust.Newspapers.Amount)	1.000	1.001	0.818	0.366840

Note. *: $p < .05$, **: $p < .01$, ***: $p < .001$, edf (Estimated Degrees of Freedom): Represents the complexity of the smooth term. A value of 1 indicates an effectively linear effect, while higher values suggest increasing non-linearity. Ref. edf (Reference Degrees of Freedom): The maximum potential degrees of freedom that the smooth term could use, based on the basis dimension specified for the term in the model.

Effects of Other Factors (not related to reading behavior)

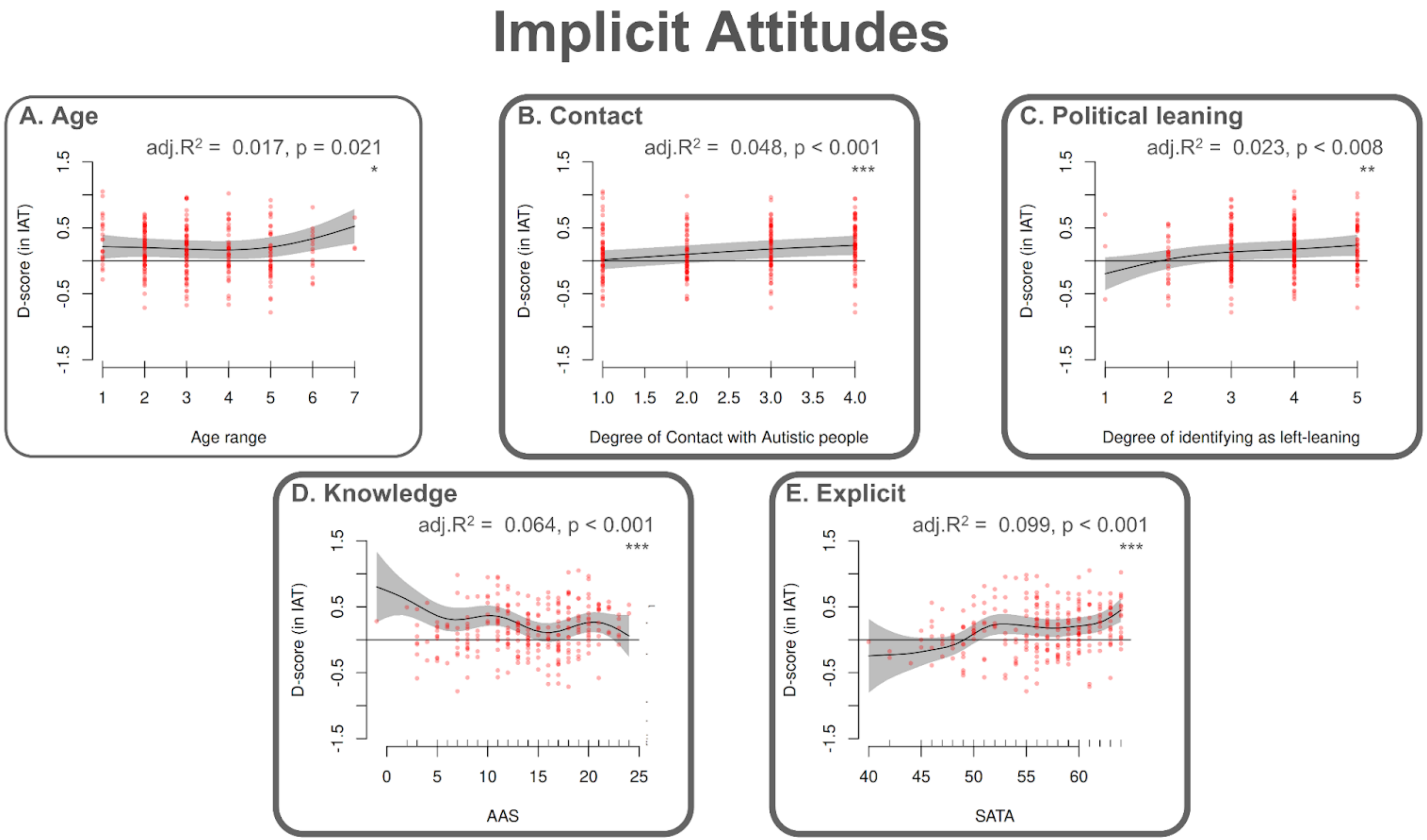

Supplementary Figure S3.

This figure illustrates the partial effects of four predictors of **implicit attitudes toward autism**, as measured with the IAT. These predictors, which are unrelated to newspaper reading preferences, are significant in the GAM model. **Panel A** shows the effect of age, **Panel B** presents the effect of prior contact with Autistic people, **Panel C** demonstrates the effect of self-reported political leaning, **Panel D** depicts the effect of knowledge about autism (measured by the AAS), and **Panel E** shows the effect of explicit attitudes (SATA score). The framing of each panel indicates the significance of the partial effects: a thin solid line signifies a significant effect, while a bold solid-line frame highlights a highly significant effect. The text at the top of each plot indicates the individual contribution to the adjusted R^2 and the corresponding p-values (*: $p < .05$, **: $p < .01$, ***: $p < .001$).

S6. Further details on the weighted GAM model for Knowledge About Autism

Rationale/Relevance to the main paper

This section presents detailed results for the parametric coefficients, which represent the categorical predictors in the GAM model for **knowledge about autism**, namely gender and political views, alongside the results for the "smooth" terms, which correspond to the non-linear predictors in the same GAM model. Additionally, **Supplementary Figure S4** depicts the partial effects of significant terms unrelated to reading preferences (for the partial effects of reading preference variables see **Figure 3** of the main paper).

Categorical Predictors

Supplementary Table S7. Estimates for Parametric coefficients (weighted GAM model for Knowledge About Autism).

	**Estimate**	**Std. Error**	**t value**	**p-value**
GENDERMale	-1.3826	0.3776	-3.661	0.000311 ***
GENDEROther	-4.4534	4.0913	-1.088	0.277510

Note. *: p < .05, **: p < .01, ***: p < .001, Std. Error: Standard Error

Non-Linear Predictors

Supplementary Table S8. Estimates for Smooth terms (weighted GAM model for Knowledge About Autism).

Term	edf	Ref.edf	F	p-value
s(AGE)	1.000	1.000	17.008	5.25e-05 ***
s(EDUCATION_YEARS)	1.000	1.000	7.013	0.008644 **
s(CONTACT)	2.192	2.552	36.221	< 2e-16 ***
s(POLITICAL)	2.948	2.995	24.482	< 2e-16 ***
s(EXPLICIT)	1.736	2.178	84.940	< 2e-16 ***
s(IMPLICIT)	3.076	3.829	6.048	0.000176 ***
s(Read.Right.Tabloids)	4.038	4.998	2.956	0.013370 *
s(Trust.Right.Tabloids)	5.289	6.498	2.016	0.057038 .
s(Trust.Newspapers.Amount)	6.736	7.974	3.746	0.000393 ***

Note. *: $p < .05$, **: $p < .01$, ***: $p < .001$, edf (Estimated Degrees of Freedom): Represents the complexity of the smooth term. A value of 1 indicates an effectively linear effect, while higher values suggest increasing non-linearity. Ref. edf (Reference Degrees of Freedom): The maximum potential degrees of freedom that the smooth term could use, based on the basis dimension specified for the term in the model.

Effects of Other Factors (not related to reading behavior)

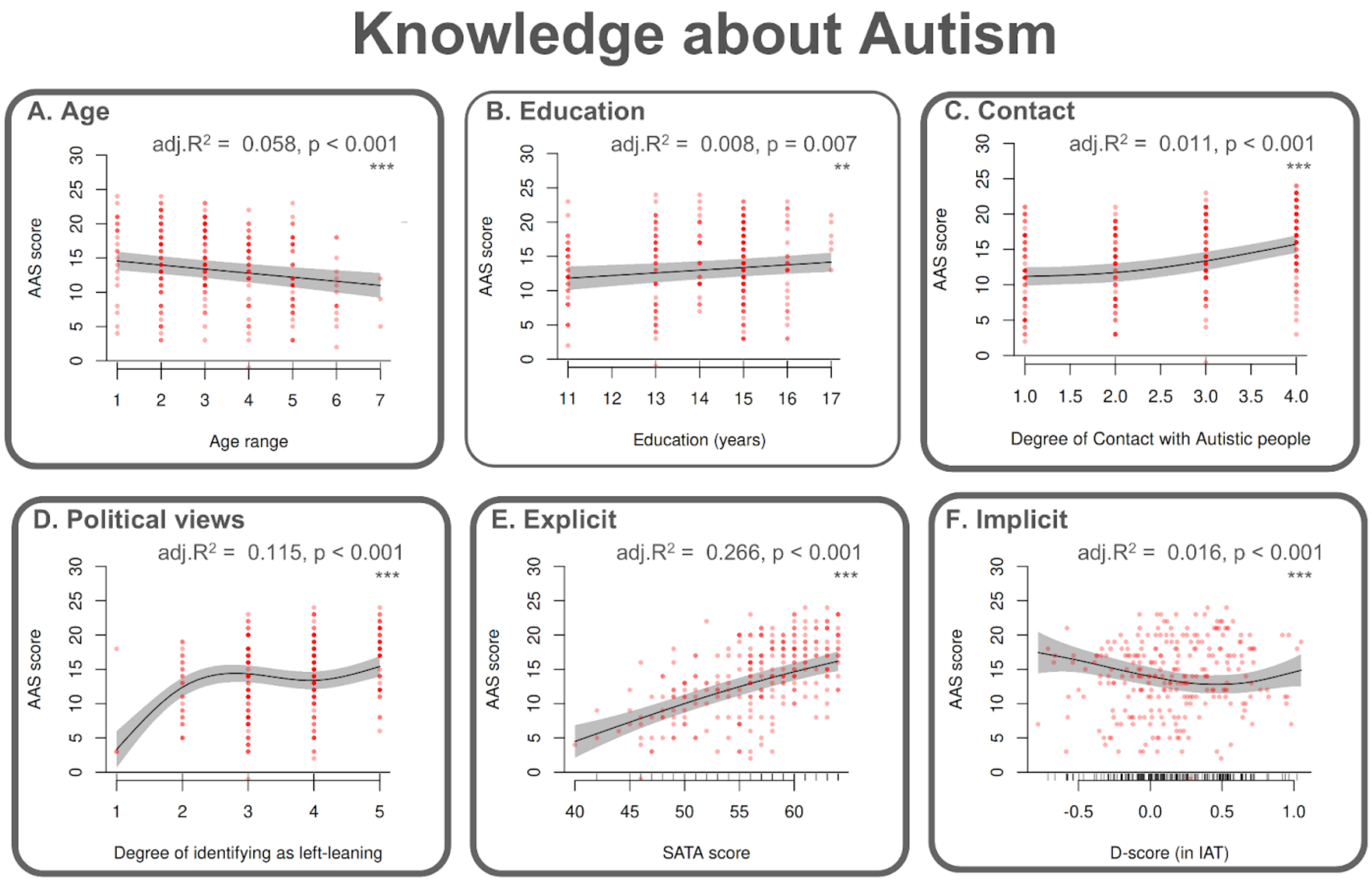

Supplementary Figure S4.

This figure illustrates the partial effects of five predictors of **knowledge about autism**, as measured with the AAS. These predictors, which are unrelated to newspaper reading preferences, are significant in the GAM model. **Panel A** shows the effect of age, **Panel B** presents the effect of educational attainment, **Panel C** illustrates the effect of prior contact with Autistic people, **Panel D** demonstrates the effect of political views, **Panel E** depicts the effect of explicit attitudes (measured with the SATA), and **Panel F** shows the effect of implicit attitudes (estimated using the D-score measure). The framing of each panel indicates the significance of the partial effects: a thin solid line signifies a significant effect, while a bold solid-line frame highlights a highly significant effect. The text at the top of each plot indicates the individual contribution to the adjusted R^2 and the corresponding p-values (*: $p < .05$, **: $p < .01$, ***: $p < .001$).

S7. Unweighted “baseline” GAM model for Explicit Attitudes

Rationale/Relevance to the main paper

This section examines the unweighted version of the GAM model for **explicit attitudes** presented in the main paper. This model does not weight data by overall exposure to newspapers; instead, this variable is one of the terms included in the GAM model.

We provide detailed results for the parametric coefficients, representing the categorical predictor in the GAM model for explicit attitudes, specifically gender, alongside the results for the "smooth" terms, which account for the non-linear predictors in the same model. Additionally, **Supplementary Figure S5** illustrates the contributions of the various factors to the explained variance in the outcome variable.

A comparison with the main model of implicit attitudes (Figure 1 in the main paper) reveals that **weighting increases the explained variance from 35.82% to 53.75%**. While political orientation, knowledge about autism, and contact with Autistic people emerge as significant predictors of explicit attitudes with a non-significant trend for education in the unweighted model, **none of the reading behavior variables is significant in the unweighted model.**

Categorical Predictors

Supplementary Table S9. Estimates for Parametric coefficients (Unweighted "baseline" GAM model for Explicit Attitudes).

	Estimate	Std. Error	t value	p-value
GENDERMale	-0.1117	0.5722	-0.195	0.845
GENDEROther	3.1745	4.3193	0.735	0.463

Note. *: p < .05, **: p < .01, ***: p < .001, Std. Error: Standard Error

Non-Linear Predictors

Supplementary Table S10. Estimates for Smooth terms (Unweighted "baseline" GAM model for Explicit Attitudes).

Term	edf	Ref.edf	F	p-value
s(AGE)	1.000	1.000	2.446	0.11909
s(EDUCATION_YEARS)	1.000	1.000	3.311	0.07004 .
s(CONTACT)	1.000	1.000	0.967	0.32633
s(POLITICAL)	1.402	1.683	0.145	0.84758
s(KNOWLEDGE)	1.000	1.001	96.106	< 2e-16 ***
s(IMPLICIT)	1.000	1.001	7.418	0.00691 **
s(Read.Right.Tabloids)	1.000	1.001	0.082	0.77518
s(Newspaper.Reading.Amount)	1.000	1.000	0.137	0.71202
s(Trust.Right.Tabloids)	2.113	2.712	1.115	0.46440
s(Trust.Newspapers.Amount)	1.003	1.005	0.087	0.77324

Note. *: p < .05, **: p < .01, ***: p < .001, edf (Estimated Degrees of Freedom): Represents the complexity of the smooth term. A value of 1 indicates an effectively linear effect, while higher

values suggest increasing non-linearity. Ref. edf (Reference Degrees of Freedom): The maximum potential degrees of freedom that the smooth term could use, based on the basis dimension specified for the term in the model.

Individual Contributions and Effects of Reading behavior

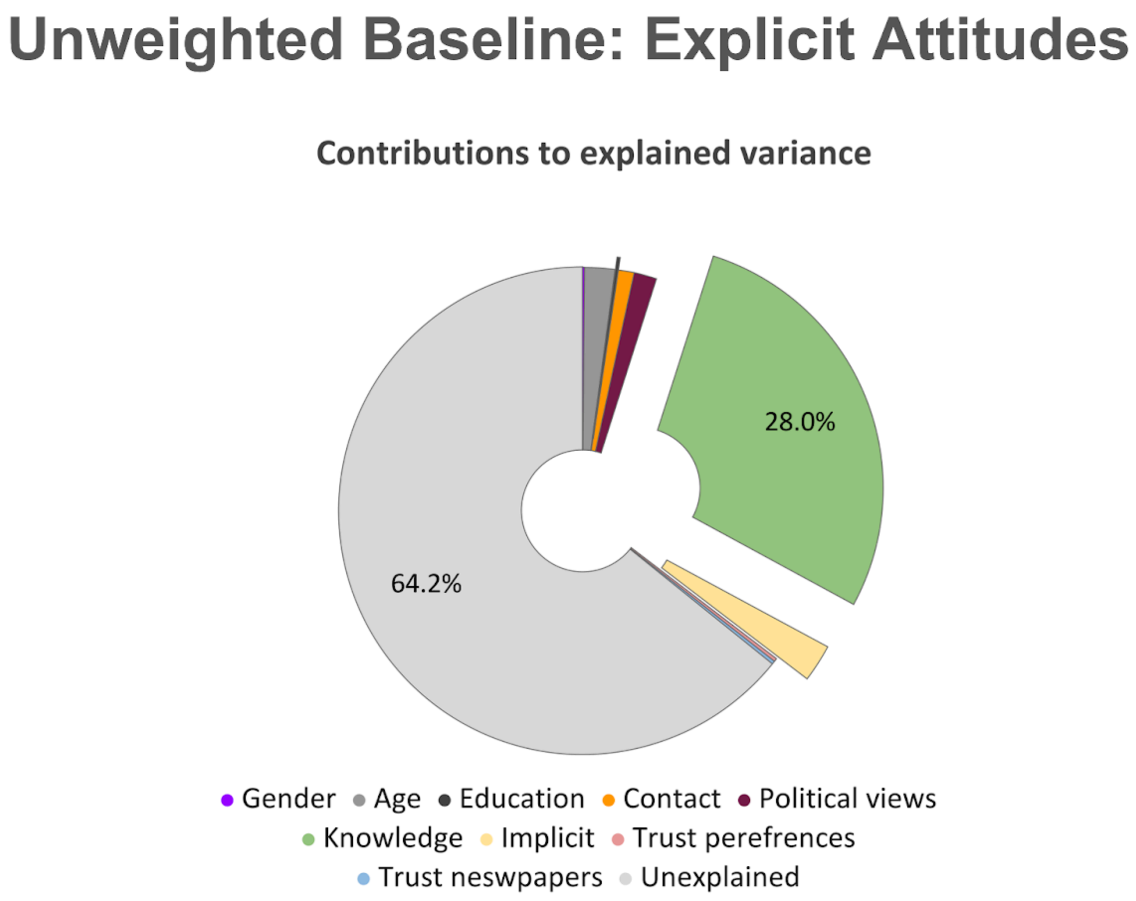

Supplementary Figure S5.

The pie chart illustrates the contribution of various predictors to the explained variance in **explicit attitudes** toward autism, as measured using the SATA. We determined these contributions using hierarchical partitioning analysis for the GAM model weighted by overall newspaper exposure. This procedure calculated the proportion of adjusted R^2 corresponding to individual predictors. The pie chart shows segments corresponding to different factors in the same order as the legend (starting from the top and moving clockwise). The distance of a segment from the centre indicates the significance of the corresponding factor (greater distance signifies lower p-values). The light gray segment represents variance that the GAM model left unexplained.

S8. Unweighted “baseline” GAM model for Implicit attitudes

Rationale/Relevance to the main paper

This section shows the **unweighted version of the GAM model for implicit attitudes** presented in the main paper. This model does not weight data by overall exposure to newspapers; instead, this variable is one of the terms included in the GAM model.

This section details the results for the parametric coefficients, which represent the categorical predictor in the GAM model for implicit attitudes, specifically gender, as well as the results for the "smooth" terms, which capture the non-linear predictors in the model. **Supplementary Figure S6** highlights the contributions of the various factors to the explained variance in the outcome variable.

A comparison with the main model of implicit attitudes (Figure 2 in the main paper) shows that **weighting increases the explained variance from 15.42% to 36.39%.** In the unweighted model, contact with Autistic people, knowledge about autism, and explicit attitudes are significant predictors of implicit attitudes, whereas **none of the reading behavior variables are significant in the unweighted model.**

Categorical Predictors

Supplementary Table S11. Estimates for Parametric coefficients (Unweighted "baseline" GAM model for Implicit attitudes).

	**Estimate**	**Std. Error**	**t value**	**p-value**
GENDERMale	-0.03710	0.04518	-0.821	0.412
GENDEROther	-0.09486	0.34096	-0.278	0.781

Note. *: p < .05, **: p < .01, ***: p < .001, Std. Error: Standard Error

Non-Linear Predictors

Supplementary Table S12. Estimates for Smooth terms (Unweighted "baseline" GAM model for Implicit attitudes).

Term	edf	Ref.edf	F	p-value
s(AGE)	2.008	2.410	1.644	0.15963
s(EDUCATION_YEARS)	1.000	1.000	0.061	0.80469
s(CONTACT)	1.000	1.000	7.516	0.00658 **
s(KNOWLEDGE)	1.573	1.908	0.816	0.50132
s(KNOWLEDGE)	5.835	7.155	2.649	0.01111 *
s(EXPILICIT)	1.000	1.000	9.111	0.00281 **
s(Read.Right.Tabloids)	1.000	1.000	2.499	0.11526
s(Newspaper.Reading.Amount)	1.000	1.000	0.013	0.91105
s(Trust.Right.Tabloids)	3.218	4.086	1.191	0.32008
s(Trust.Newspapers.Amount)	1.536	1.909	0.300	0.71210

Note. *: $p < .05$, **: $p < .01$, ***: $p < .001$, edf (Estimated Degrees of Freedom): Represents the complexity of the smooth term. A value of 1 indicates an effectively linear effect, while higher values suggest increasing non-linearity. Ref. edf (Reference Degrees of Freedom): The maximum potential degrees of freedom that the smooth term could use, based on the basis dimension specified for the term in the model.

Individual Contributions

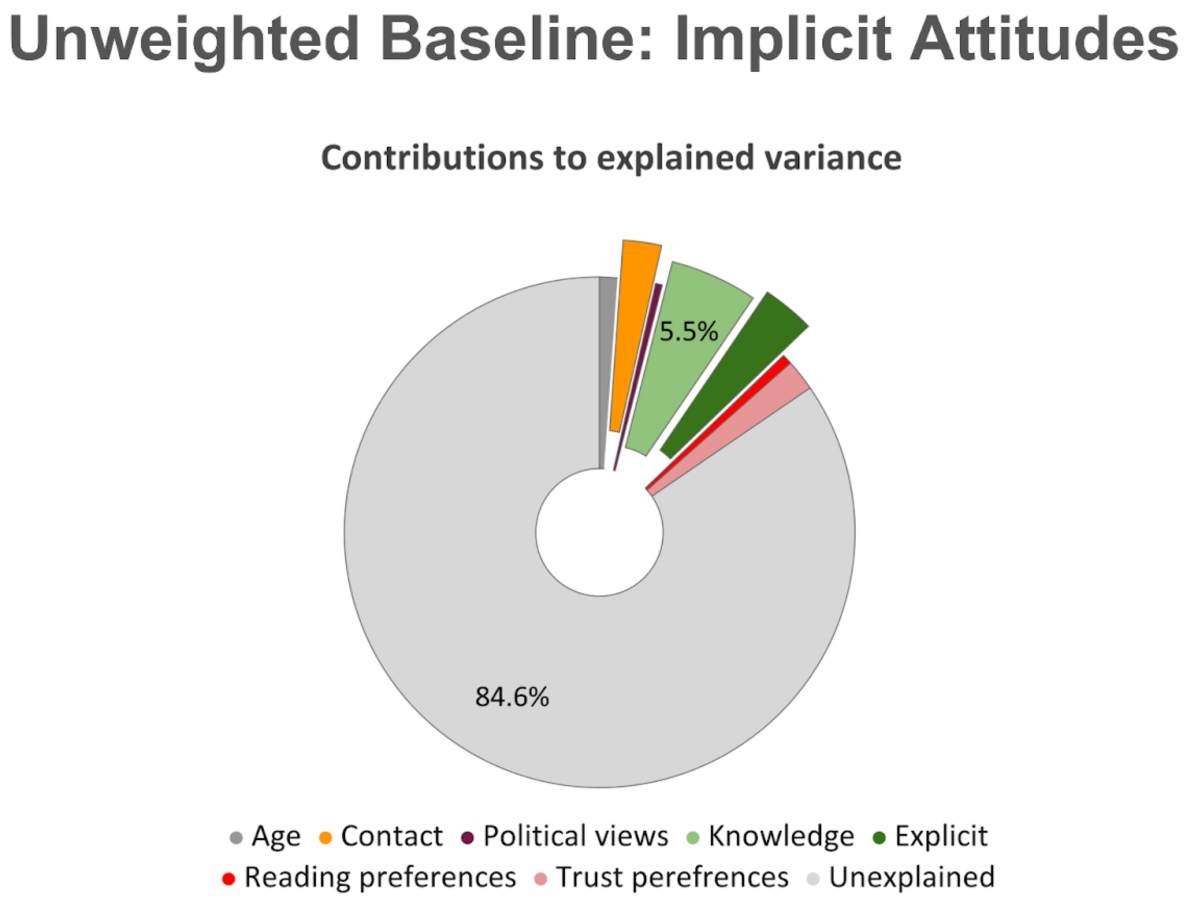

Supplementary Figure S6

The pie chart illustrates the contribution of various predictors to the explained variance in implicit attitudes toward autism, as measured by the SC-IAT. We determined these contributions using hierarchical partitioning analysis for the GAM model weighted by overall newspaper exposure. This procedure calculated the proportion of adjusted R^2 corresponding to individual predictors. The pie chart shows segments corresponding to different factors in the same order as the legend (starting from the top and moving clockwise). The distance of a segment from the centre indicates the significance of the corresponding factor (greater distance signifies lower p-values). The light gray segment represents variance that the GAM model left unexplained.

S9. Unweighted “baseline” GAM model for Knowledge about Autism

Rationale/Relevance to the main paper

This section shows the **unweighted version of the GAM model for knowledge about autism** presented in the main paper. This model does not weight data by overall exposure to newspapers; instead, this variable is one of the terms included in the GAM model.

This section details the results for the parametric coefficients, which represent the categorical predictor in the GAM model for knowledge about autism, specifically gender, as well as the results for the "smooth" terms, which capture the non-linear predictors in the model. **Supplementary Figure S7** highlights the contributions of the various factors to the explained variance in the outcome variable.

A comparison with the main model of knowledge about autism (Figure 3 in the main paper) shows that **weighting increases the explained variance from 46.62% to 70.25%.** In the unweighted model, political orientation, contact with Autistic people, and explicit attitudes are significant predictors of knowledge about autism (with a non-significant trend for an effect of gender), whereas none of the reading behavior variables are significant.

Categorical Predictors

Supplementary Table S13. Estimates for Parametric coefficients (Unweighted "baseline" GAM model for Knowledge about Autism).

	**Estimate**	**Std. Error**	**t value**	**p-value**
GENDERMale	-0.9260	0.5145	-1.80	0.0731 .
GENDEROther	-1.9534	3.9098	-0.50	0.6178

Note. . : p < 0.1 (non-significant trend); *: p < .05, **: p < .01, ***: p < .001, Std. Error: Standard Error

Non-Linear Predictors

Supplementary Table S14. Estimates for Smooth terms (Unweighted "baseline" GAM model for Knowledge about Autism).

Term	edf	Ref.edf	F	p-value
s(AGE)	1.000	1.000	1.595	0.20777
s(EDUCATION_YEARS)	1.000	1.000	1.576	0.21048
s(CONTACT)	1.165	1.307	19.766	7.24e-06 ***
s(POLITICAL)	1.000	1.000	10.641	0.00126 **
s(EXPLICIT)	1.000	1.000	97.245	< 2e-16 ***
s(IMPLICIT)	1.000	1.001	1.233	0.26781
s(Read.Right.Tabloids)	2.583	3.392	0.601	0.54352
s(Newspaper.Reading.Amount)	1.000	1.001	0.028	0.86875
s(Trust.Right.Tabloids)	2.358	3.027	1.259	0.29647
s(Trust.Newspapers.Amount)	1.000	1.000	2.469	0.11736

Note. *: p < .05, **: p < .01, ***: p < .001, edf (Estimated Degrees of Freedom): Represents the complexity of the smooth term. A value of 1 indicates an effectively linear effect, while higher values suggest increasing non-linearity. Ref. edf (Reference Degrees of Freedom): The maximum potential degrees of freedom that the smooth term could use, based on the basis dimension specified for the term in the model.

Individual Contributions

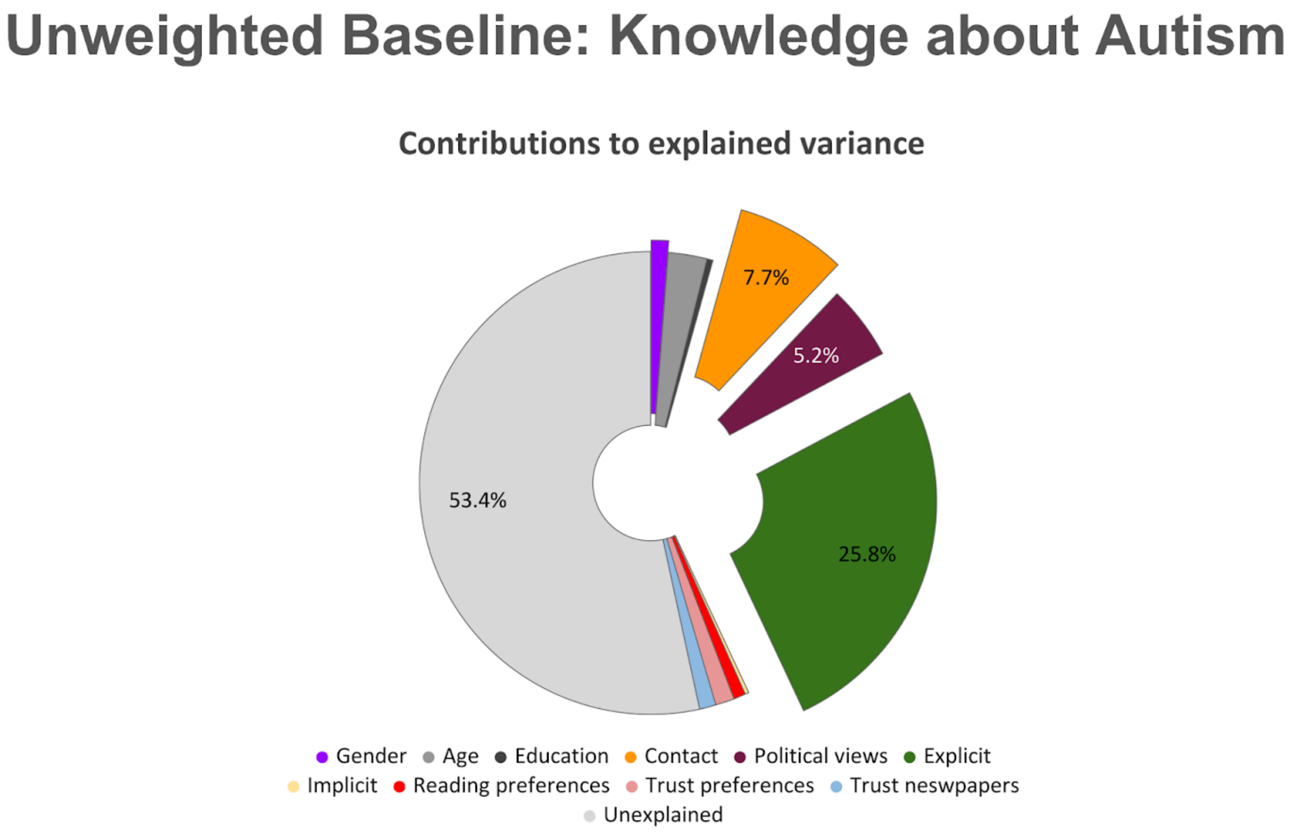

Supplementary Figure S7

The pie chart illustrates the contribution of various predictors to the explained variance in knowledge about autism, as assessed with the AAS. We determined these contributions using hierarchical partitioning analysis for the GAM model weighted by overall newspaper exposure. This procedure calculated the proportion of adjusted R^2 corresponding to individual predictors. The pie chart shows segments corresponding to different factors in the same order as the legend (starting at the top and moving clockwise). The distance of a segment from the centre indicates the significance of the corresponding factor (greater distance signifies lower p-values). The light gray segment represents variance that the GAM model left unexplained.

S10. Alternative weighted GAM model for Explicit Attitudes: All data (including "Other" and "Prefer not to say" responses for political leaning)

Rationale/Relevance to the main paper

In the results reported in the main paper, political orientation was analysed as a scale after removing "Other" (N = 3) and "Prefer not to say" (N = 12) responses. However, we also conducted control analyses in which we included these "Other" and "Prefer not to say" responses and **treated political orientation as a categorical factor without order.**

This section reports these control analyses for **explicit attitudes**. **Supplementary Tables S15 and S16** provide the GAM model's summary of effects, while **Supplementary Figure S8** has the same layout as **Figure 2 i**n the main paper. We find a similar pattern of results on the contribution of the different factors to explicit attitudes, suggesting that the decision to exclude this data did not affect the findings reported in the paper.

Categorical Predictors

Supplementary Table S15. Estimates for Parametric coefficients (weighted GAM model for Explicit Attitudes using all the data, i.e., including "Other" and "Prefer not to say" responses for political leaning).

	**Estimate**	**Std. Error**	**t value**	**p-value**
GENDERMale	1.6355	0.5209	3.140	0.0019 **
GENDEROther	1.5614	5.3019	0.295	0.7686
POLITICAL2	-10.7759	2.3122	-4.661	5.17e-06 ***
POLITICAL3	-10.6945	2.2171	-4.824	2.47e-06 ***
POLITICAL4	-10.9137	2.2292	-4.896	1.77e-06 ***
POLITICAL5	-10.3640	2.3450	-4.420	1.48e-05 ***
POLITICAL7	-10.3427	6.0350	-1.714	0.0878 .
POLITICAL8	-10.4911	2.3038	-4.554	8.29e-06 ***

Note. *: $p < .05$, **: $p < .01$, ***: $p < .001$, Std. Error: Standard Error. POLITICAL1 (reference): Right; POLITICAL2: Right-leaning; POLITICAL3: Center; POLITICAL4: Left-leaning; POLITICAL5: Left; POLITICAL7: Other; POLITICAL8: Prefer not to say.

Non-Linear Predictors

Supplementary Table S16. Estimates for Smooth terms (weighted GAM model for Explicit Attitudes using all the data, i.e., including "Other" and "Prefer not to say" responses for political leaning).

Term	edf	Ref.edf	F	p-value
s(AGE)	2.186	2.581	2.157	0.0891 .
s(EDUCATION_YEARS)	1.000	1.000	6.442	0.0118 *
s(CONTACT)	1.000	1.001	15.751	9.5e-05 ***
s(KNOWLEDGE)	4.811	5.964	35.075	< 2e-16 ***
s(IMPLICIT)	3.735	4.628	10.956	< 2e-16 ***
s(Read.Right.Tabloids)	2.749	3.500	1.633	0.1764
s(Trust.Right.Tabloids)	5.129	6.327	2.739	0.0145 *
s(Trust.Newspapers.Amount)	1.001	1.001	1.872	0.1724

Note. *: $p < .05$, **: $p < .01$, ***: $p < .001$, edf (Estimated Degrees of Freedom): Represents the complexity of the smooth term. A value of 1 indicates an effectively linear effect, while higher values suggest increasing non-linearity. Ref. edf (Reference Degrees of Freedom): The maximum potential degrees of freedom that the smooth term could use, based on the basis dimension specified for the term in the model.

Individual Contributions and Effects of Reading behavior

All data: Explicit Attitudes

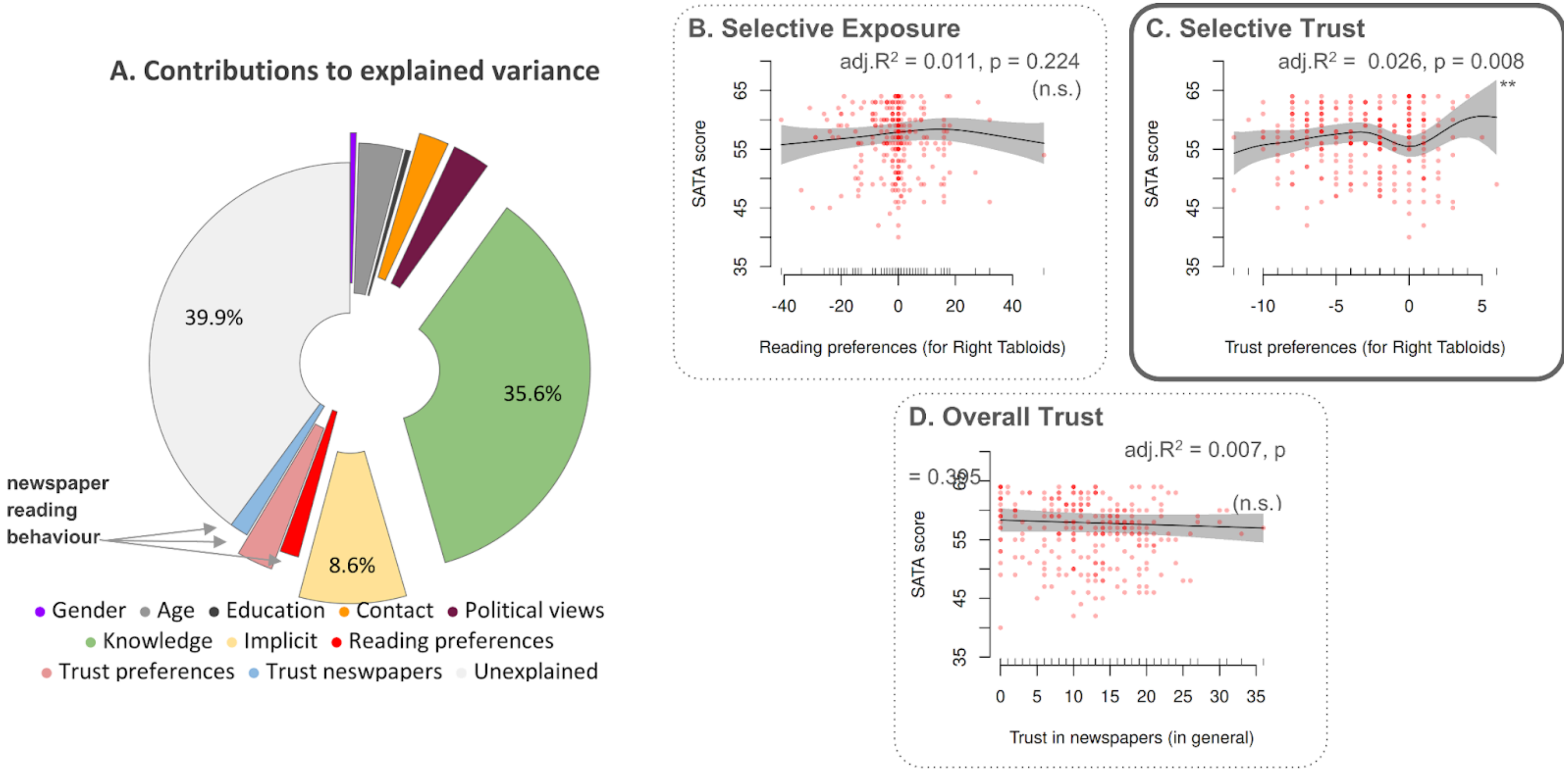

Supplementary Figure S8.

A. The pie chart illustrates the contribution of various predictors to the variance that the GAM model for explicit attitudes explains. **B, C, and D.** Panels depict the partial effects of three predictors relevant to newspaper reading preferences on explicit attitudes.The configuration of this figure is identical to **Figure 1;** the reader may refer to it for further details and for comparison.

S11. Alternative weighted GAM model for Implicit Attitudes using all the data (including "Other" and "Prefer not to say" responses for political leaning).

Rationale/Relevance to the main paper

This section presents the control analyses for **implicit attitudes,** including responses from participants who identified their political orientation as "Other" or "prefer not to say." The results of the GAM models are summarized in **Supplementary Tables S16 and S17**, while **Supplementary Figure S9** mirrors the layout of **Figure 3** in the main manuscript. We observed a comparable pattern in how different factors contributed to explicit attitudes, indicating that excluding this data did not alter the conclusions presented in the paper.

Categorical Predictors

Supplementary Table S17. Estimates for Parametric coefficients (weighted GAM model for Implicit Attitudes using all the data, i.e., including "Other" and "Prefer not to say" responses for political leaning).

	**Estimate**	**Std. Error**	**t value**	**p-value**
GENDERMale	-0.08864	0.04204	-2.108	0.03601 *
GENDEROther	-0.19072	0.41108	-0.464	0.64310
POLITICAL2	0.35775	0.18997	1.883	0.06086 .
POLITICAL3	0.47479	0.18239	2.603	0.00980 **
POLITICAL4	0.48077	0.18457	2.605	0.00975 **
POLITICAL5	0.56216	0.19058	2.950	0.00349 **
POLITICAL7	0.30961	0.47139	0.657	0.51193
POLITICAL8	0.39546	0.19240	2.055	0.04089 *

Note. *: $p < .05$, **: $p < .01$, ***: $p < .001$, Std. Error: Standard Error. POLITICAL1 (reference): Right; POLITICAL2: Right-leaning; POLITICAL3: Center; POLITICAL4: Left-leaning; POLITICAL5: Left; POLITICAL7: Other; POLITICAL8: Prefer not to say.

Non-Linear Predictors

Supplementary Table S18. Estimates for Smooth terms (Estimates for Parametric coefficients (weighted GAM model for Implicit Attitudes using all the data, i.e., including "Other" and "Prefer not to say" responses for political leaning).

Term	edf	Ref.edf	F	p-value
s(AGE)	2.519	2.830	4.263	0.018840 *
s(EDUCATION_YEARS)	1.610	1.950	0.665	0.462764
s(CONTACT)	1.000	1.000	17.099	4.94e-05 ***
s(KNOWLEDGE)	5.229	6.070	4.026	0.000712 ***
s(EXPLICIT)	4.372	5.431	7.005	2.90e-06 ***
s(Read.Right.Tabloids)	1.000	1.000	17.579	3.90e-05 ***
s(Trust.Right.Tabloids)	4.858	6.033	3.020	0.007084 **
s(Trust.Newspapers.Amount)	1.000	1.000	0.898	0.344169

Note. *: $p < .05$, **: $p < .01$, ***: $p < .001$, edf (Estimated Degrees of Freedom): Represents the complexity of the smooth term. A value of 1 indicates an effectively linear effect, while higher values suggest increasing non-linearity. Ref. edf (Reference Degrees of Freedom): The maximum potential degrees of freedom that the smooth term could use, based on the basis dimension specified for the term in the model.

Individual Contributions and Effects of Reading Behavior

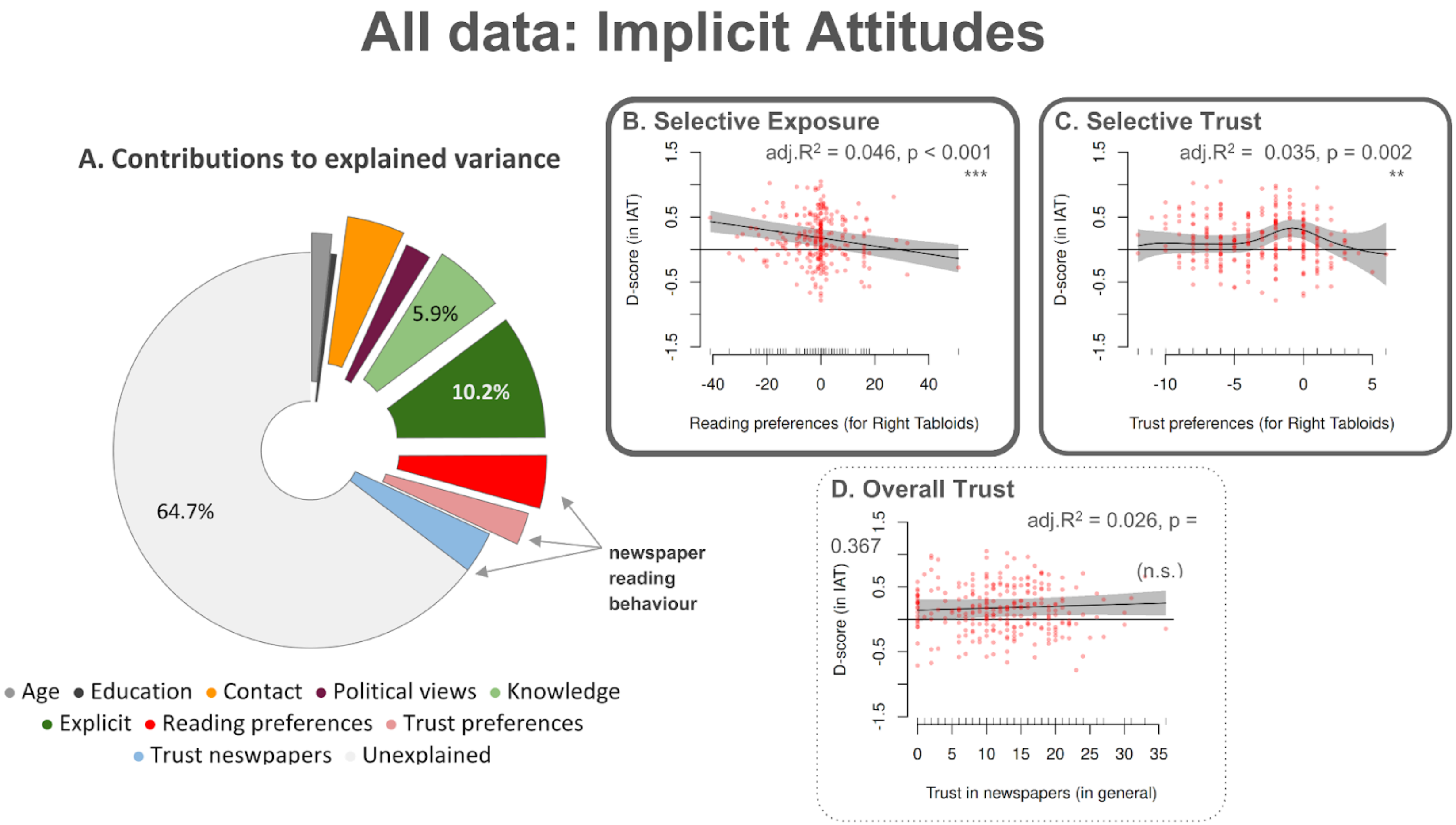

Supplementary Figure S9

A. The pie chart illustrates the contribution of various predictors to the variance that the GAM model for implicit attitudes explains. **B, C, and D**. Panels depict the partial effects of three predictors relevant to newspaper reading preferences on implicit attitudes.The configuration of this figure is identical to **Figure 2;** the reader may refer to it for further details and for comparison.

S12. Alternative weighted GAM model for Knowledge about Autism using all the data (including "Other" and "Prefer not to say" responses for political leaning).

Rationale/Relevance to the main paper

This section outlines control analyses of autism knowledge, including participants who reported their political orientation as "Other" or "Prefer not to say." **Supplementary Tables S18 and S19** summarize the GAM model outputs, while **Supplementary Figure S10** maintains the same structure as **Figure 4** in the main text. We found a similar pattern in the contributions of various factors to explicit attitudes, implying that excluding these participants' data did not alter the study's overall results.

.

Categorical Predictors

Supplementary Table S19. Estimates for Parametric coefficients (weighted GAM model for Knowledge about Autism using all the data, i.e., including "Other" and "Prefer not to say" responses for political leaning).

	**Estimate**	**Std. Error**	**t value**	**p-value**
GENDERMale	-1.5323	0.3867	-3.962	9.72e-05 ***
GENDEROther	-3.3882	4.1014	-0.826	0.410
POLITICAL2	13.2564	1.6142	8.213	1.19e-14 ***
POLITICAL3	13.4864	1.5164	8.893	< 2e-16 ***
POLITICAL4	13.4755	1.5472	8.710	4.36e-16 ***
POLITICAL5	15.0088	1.5974	9.396	< 2e-16 ***
POLITICAL7	6.3062	4.6959	1.343	0.181
POLITICAL8	12.4278	1.7081	7.276	4.50e-12 ***

Note. . : $p < 0.1$ (non-significant trend); *: $p < .05$, **: $p < .01$, ***: $p < .001$, Std. Error: Standard Error. POLITICAL1 (reference): Right; POLITICAL2: Right-leaning; POLITICAL3: Center; POLITICAL4: Left-leaning; POLITICAL5: Left; POLITICAL7: Other; POLITICAL8: Prefer not to say.

Non-Linear Predictors

Supplementary Table S20. Estimates for Smooth terms (weighted GAM model for Knowledge about Autism using all the data, i.e., including "Other" and "Prefer not to say" responses for political leaning).

Term	edf	Ref.edf	F	p-value
s(AGE)	1.000	1.001	21.630	8.66e-07 ***
s(EDUCATION_YEARS)	1.000	1.001	5.840	0.01636 *
s(CONTACT)	2.249	2.609	35.071	< 2e-16 ***
s(EXPLICIT)	2.146	2.702	69.990	< 2e-16 ***
s(IMPLICIT)	3.124	3.903	5.064	0.00086 ***
s(Read.Right.Tabloids)	3.598	4.496	2.471	0.04467 *
s(Trust.Right.Tabloids)	2.086	2.653	1.387	0.27892
s(Trust.Newspapers.Amount)	4.454	5.474	2.706	0.03139 *

Note. *: $p < .05$, **: $p < .01$, ***: $p < .001$, edf (Estimated Degrees of Freedom): Represents the complexity of the smooth term. A value of 1 indicates an effectively linear effect, while higher values suggest increasing non-linearity. Ref. edf (Reference Degrees of Freedom): The maximum potential degrees of freedom that the smooth term could use, based on the basis dimension specified for the term in the model.

Individual Contributions and Effects of Reading Behavior

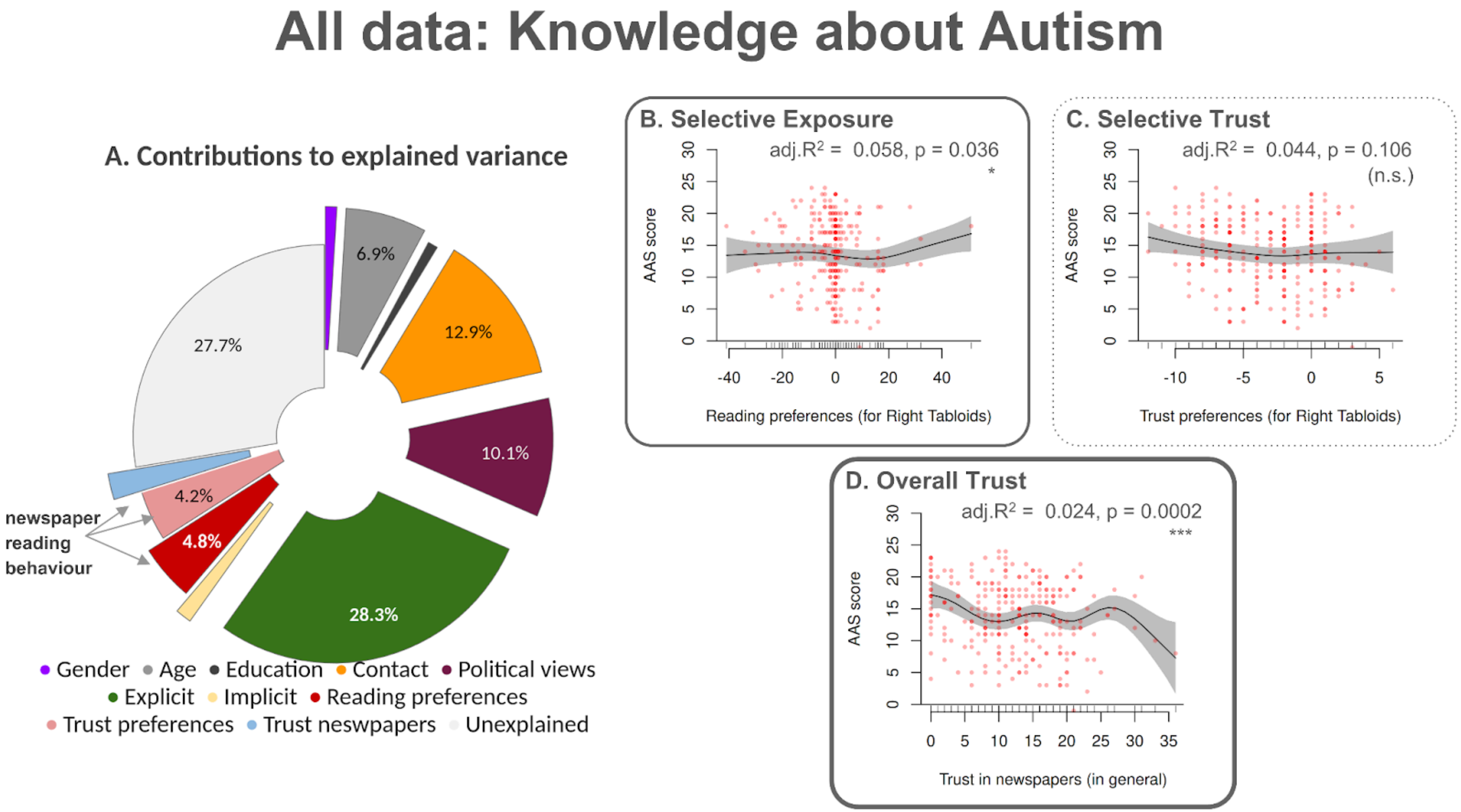

Supplementary Figure S10

A. The pie chart displays the relative contributions of different predictors to the variance explained by the GAM model for knowledge about autism. **B, C, and D.** These panels show the partial effects of three newspaper-reading preference predictors on knowledge about autism. The layout and styling mirror those in **Figure 4**, and readers are directed to that figure for more detailed interpretation.

S13. Alternative unweighted GAM model for Explicit Attitudes: Reduced dataset (excluding participants with minimal exposure to newspapers)

Rationale/Relevance to the main paper

This section shows an alternative GAM model for **explicit attitudes**, where **instead of weighting the data by overall exposure to newspapers, we applied a cut-off value.** The model uses a reduced dataset, excluding 73 participants with minimal exposure scores (≤3).

It provides detailed results for the parametric coefficients, representing the categorical predictor in the GAM model for explicit attitudes, specifically gender, along with the results for the "smooth" terms, which account for the non-linear predictors in the model. **Supplementary Figure S11** illustrates the contributions of the various factors to the explained variance in the outcome variable.

This model accounts for 43.15% of the variance—less than the 53.75% explained by the weighted GAM in the main paper, but greater than the 35.82% captured by the unweighted baseline model (see **Supplementary Section S7**). These results suggest that excluding participants with minimal newspaper engagement improves model performance, though not as much as applying weights.

In this reduced dataset model, knowledge about autism and implicit attitudes remain significant predictors, showing similar effects to those observed in the main analysis. Among the reading behavior variables, only trust in right-leaning tabloids contributes to the explained variance, though this effect is not statistically significant. Thus, **the effect of selective trust in right leaning tabloids shown in the weighted GAM model presented in the main paper, does not hold for this alternative "reduced dataset" model.**

Categorical Predictors

Supplementary Table S21. Estimates for Parametric coefficients (unweighted GAM model for Explicit Attitudes fitted to the reduced dataset).

	**Estimate**	**Std. Error**	**t value**	**p-value**
GENDERMale	0.03636	0.63231	0.058	0.954
GENDEROther	3.32171	4.18971	0.793	0.429

Note. . : p < 0.1 (non-significant trend); *: p < .05, **: p < .01, ***: p < .001

Non-Linear Predictors

Supplementary Table S22. Estimates for Smooth terms (unweighted GAM model for Explicit Attitudes fitted to the reduced dataset).

Term	edf	Ref.edf	F	p-value
s(AGE)	1.475	1.787	0.377	0.708035
s(EDUCATION_YEARS)	1.337	1.589	1.034	0.250007
s(CONTACT)	1.000	1.000	3.375	0.067848 .
s(POLITICAL)	1.123	1.233	0.011	0.965828
s(KNOWLEDGE)	2.216	2.797	35.451	< 2e-16 ***
s(IMPLICIT)	2.101	2.666	8.190	0.000106 ***
s(Read.Right.Tabloids)	1.001	1.002	0.079	0.780234
s(Newspaper.Reading.Amount)	1.000	1.000	0.108	0.742935
s(Trust.Right.Tabloids)	2.423	3.102	1.008	0.402830
s(Trust.Newspapers.Amount)	1.250	1.461	0.305	0.780983

Note. *: p < .05, **: p < .01, ***: p < .001

Individual Contributions and Effects of Reading behavior

Reduced Dataset (Unweighted): Explicit Attitudes

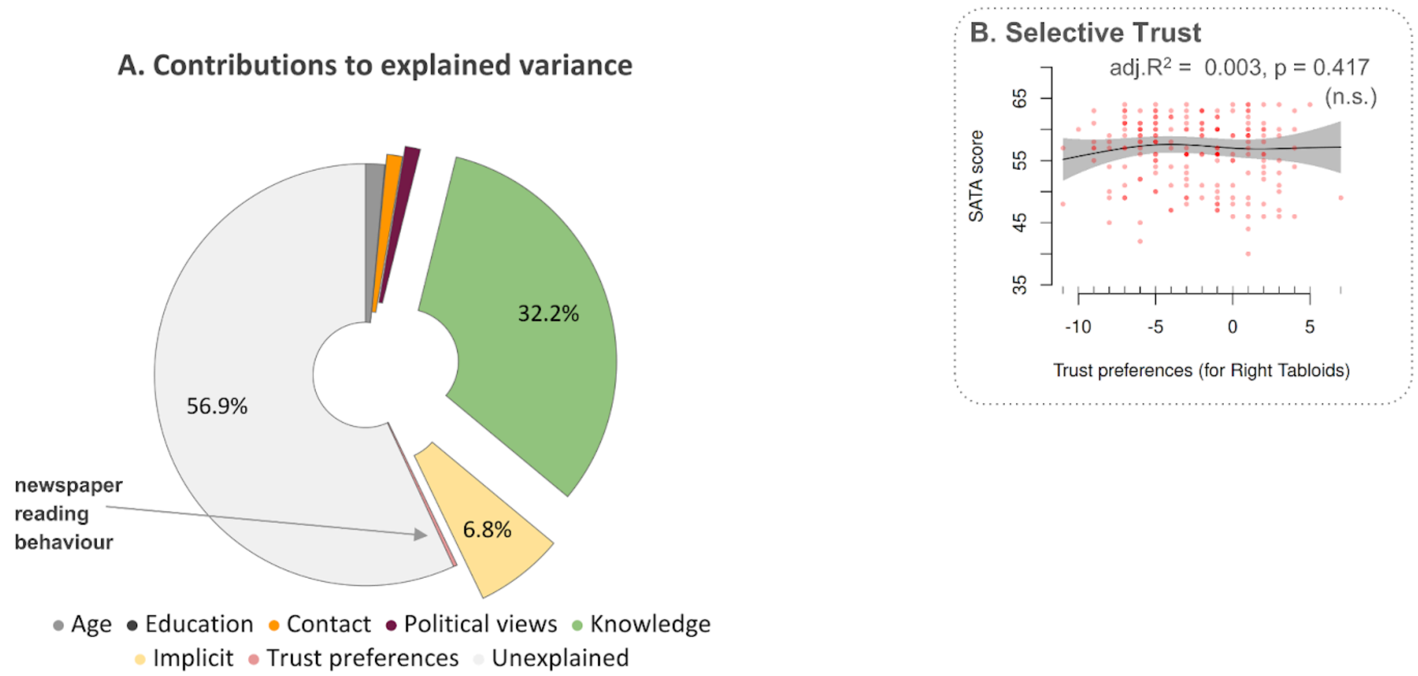

Supplementary Figure S11.

A. The pie chart illustrates the contribution of various predictors to the variance that the GAM model for explicit attitudes explains. **B.** This panel depicts the partial effects of selective trust in right-leaning tabloids, the only predictor relevant to newspaper reading behavior that contributes to explained variance in explicit attitudes. The configuration of this figure is identical to Figure 1; the reader may refer to it for further details and for comparison.

S14. Alternative unweighted GAM model for Implicit Attitudes: Reduced dataset (excluding participants with minimal exposure to newspapers)

Rationale/Relevance to the main paper

This section presents an alternative **GAM model for implicit attitudes, in which a cut-off was applied to the newspaper exposure measure instead of using exposure-based weighting**. The analysis excludes 73 participants with minimal exposure scores (≤3), resulting in a reduced dataset.

The model output includes detailed results for the parametric term (gender) as well as the smooth terms that model non-linear effects. **Supplementary Figure S12** illustrates the contribution of each predictor to the variance explained in implicit attitudes.

This version of the model explains 25.69% of the variance— less than the 36.39% accounted for by the weighted GAM in the main paper, but considerably more than the 15.42% explained by the unweighted baseline model (see **Supplementary Section S8**). These findings indicate that **filtering out participants with minimal newspaper engagement enhances the model's performance, though not as effectively as applying exposure weights.**

In this reduced dataset, significant predictors include prior contact with Autistic individuals, knowledge about autism, and explicit attitudes, all consistent with findings from the primary model. **Among reading behavior variables, selective exposure continues to show a linear effect, aligning with results reported in the main analysis. The model also captures a non-linear effect of selective trust, suggesting the robustness of the key findings regarding media-related predictors of implicit attitudes.**

Categorical Predictors

Supplementary Table 23. Estimates for Parametric coefficients (unweighted GAM model for Implicit Attitudes fitted to the reduced dataset).

	Estimate	Std. Error	t value	p-value
GENDERMale	-0.06515	0.04795	-1.359	0.176
GENDEROther	-0.16242	0.31845	-0.510	0.611

Note. *: p < .05, **: p < .01, ***: p < .001, Std. Error: Standard Error

Non-Linear Predictors

Supplementary Table S24. Estimates for Smooth terms (unweighted GAM model for Implicit Attitudes fitted to the reduced dataset)..

Term	edf	Ref.edf	F	p-value
s(AGE)	2.159	2.550	1.670	0.132355
s(EDUCATION_YEARS)	1.548	1.883	0.416	0.621603
s(CONTACT)	1.000	1.000	12.398	0.000548 ***
s(POLITICAL)	1.437	1.723	2.058	0.217147
s(KNOWLEDGE)	4.930	6.121	3.056	0.006619 **
s(EXPLICIT)	1.000	1.000	23.259	3.29e-06 ***
s(Read.Right.Tabloids)	1.000	1.000	5.484	0.020317 *
s(Newspaper.Reading.Amount)	1.000	1.000	0.106	0.745345
s(Trust.Right.Tabloids)	4.173	5.235	2.428	0.042884 *
s(Trust.Newspapers.Amount)	1.000	1.000	0.456	0.500436

Note. *: p < .05, **: p < .01, ***: p < .001, edf (Estimated Degrees of Freedom): Represents the complexity of the smooth term. A value of 1 indicates an effectively linear effect, while higher values suggest increasing non-linearity. Ref. edf (Reference Degrees of Freedom): The maximum potential degrees of freedom that the smooth term could use, based on the basis dimension specified for the term in the model.

Individual Contributions and Effects of Reading behavior

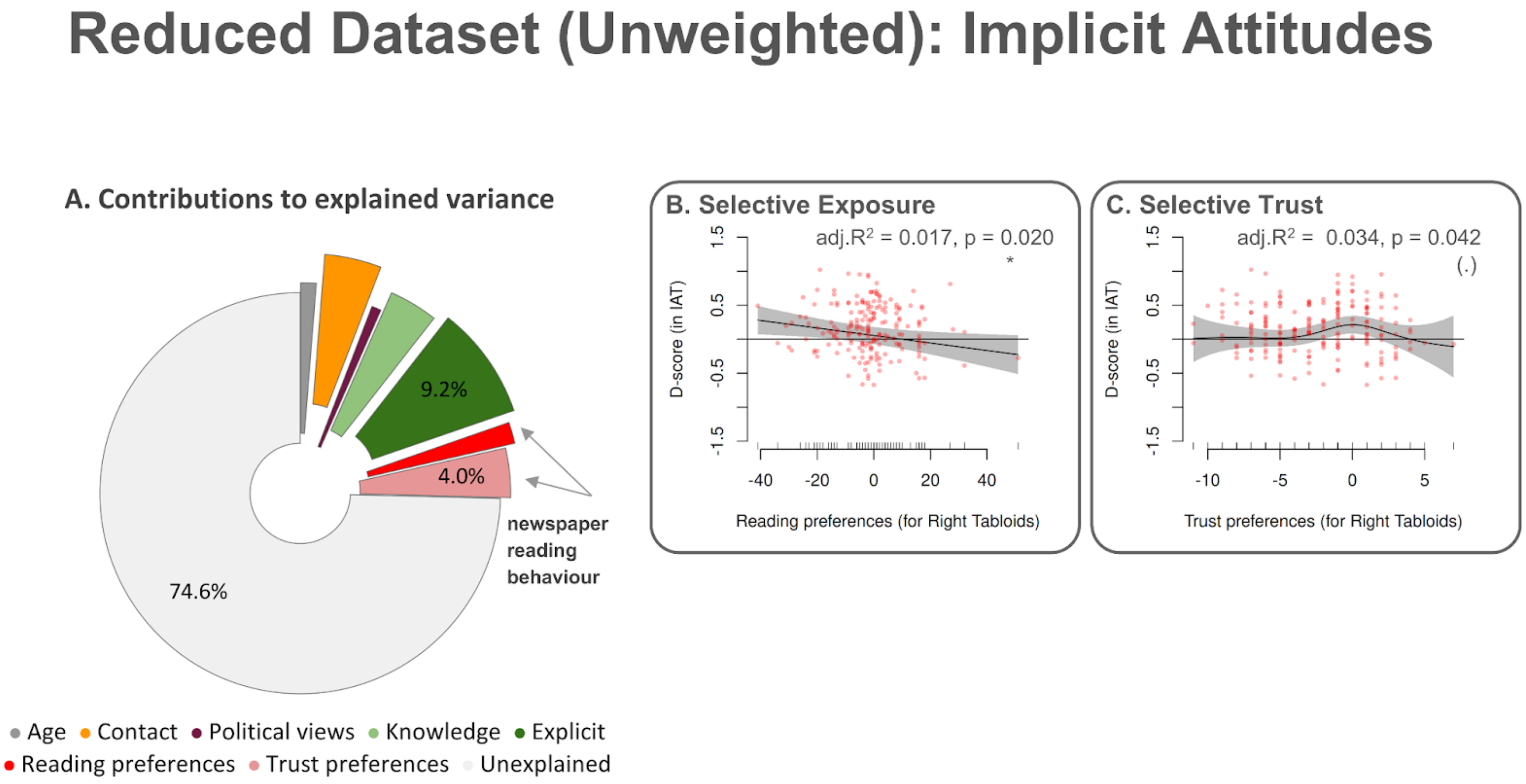

Supplementary Figure S12

A. The pie chart illustrates the contributions of various predictors to the explained variance in implicit attitudes toward autism, as measured by the SC-IAT.

B and C. These panels show the partial effects of two predictors related to newspaper reading preferences on implicit attitudes: selective exposure and selective trust. Overall trust and overall exposure are not displayed, as they contributed zero to the explained variance.

The configuration of this figure is identical to **Figure 2**; the reader may refer to its caption for further details.

S15. Alternative unweighted GAM model for Knowledge about Autism: Reduced dataset (excluding participants with minimal exposure to newspapers)

Rationale/Relevance to the main paper

This section presents an alternative GAM model for **knowledge about autism**, where we applied **a cut-off instead of weighting the data by overall exposure to newspapers**. The model uses a reduced dataset, excluding 73 participants with minimal exposure scores (≤3).

It provides detailed results for the parametric coefficients, representing the categorical predictor in the GAM model for knowledge about autism (gender), alongside the results for the "smooth" terms, which capture the non-linear predictors in the model. **Supplementary Figure S13** illustrates the contributions of the various factors to the explained variance in the outcome variable.

This version of the model explains 54.73% of the variance— less than the 70.25% accounted for by the weighted GAM in the main paper, but considerably more than the 46.62% explained by the unweighted baseline model (see **Supplementary Section S9**). These findings indicate that filtering out participants with minimal newspaper engagement enhances the model's performance, though not as effectively as applying exposure weights.

In this reduced-dataset GAM model, prior contact with Autistic people, explicit attitudes, implicit attitudes, and years of education all emerge as significant predictors—effects that are consistent with those from the main model. However, none of the newspaper reading behavior variables are significant predictors of knowledge about autism in this unweighted alternative analysis. As a result, this version of the model **does not demonstrate an observed effect in the main model, where greater trust in newspapers was associated with poorer autism knowledge.**

Categorical Predictors

Supplementary Table S25. Estimates for Parametric coefficients (unweighted GAM model for Implicit Attitudes fitted to the reduced dataset).

	**Estimate**	**Std. Error**	**t value**	**p-value**
GENDERMale	-0.8684	0.5448	-1.594	0.113
GENDEROther	-3.1136	3.6424	-0.855	0.394

Note. *: p < .05, **: p < .01, ***: p < .001, Std. Error: Standard Error

Non-Linear Predictors

Supplementary Table S26. Estimates for Smooth terms (unweighted GAM model for Implicit Attitudes fitted to the reduced dataset).

Term	edf	Ref.edf	F	p-value
s(AGE)	1.000	1.000	5.954	0.0157 *
s(EDUCATION_YEARS)	1.269	1.484	1.913	0.1124
s(CONTACT)	2.156	2.532	15.344	3.12e-07 ***
s(POLITICAL)	1.000	1.000	3.573	0.0603 .
s(EXPLICIT)	1.000	1.001	96.445	< 2e-16 ***
s(IMPLICIT)	2.079	2.635	3.822	0.0160 *
s(Read.Right.Tabloids)	2.980	3.873	0.877	0.4018
s(Newspaper.Reading.Amount)	1.000	1.001	1.092	0.2975
s(Trust.Right.Tabloids)	2.358	3.027	1.386	0.2543
s(Trust.Newspapers.Amount)	1.000	1.000	2.367	0.1257

Note. *: $p < .05$, **: $p < .01$, ***: $p < .001$, edf (Estimated Degrees of Freedom): Represents the complexity of the smooth term. A value of 1 indicates an effectively linear effect, while higher values suggest increasing non-linearity. Ref. edf (Reference Degrees of Freedom): The maximum potential degrees of freedom that the smooth term could use, based on the basis dimension specified for the term in the model.

Individual Contributions and Effects of Reading behavior

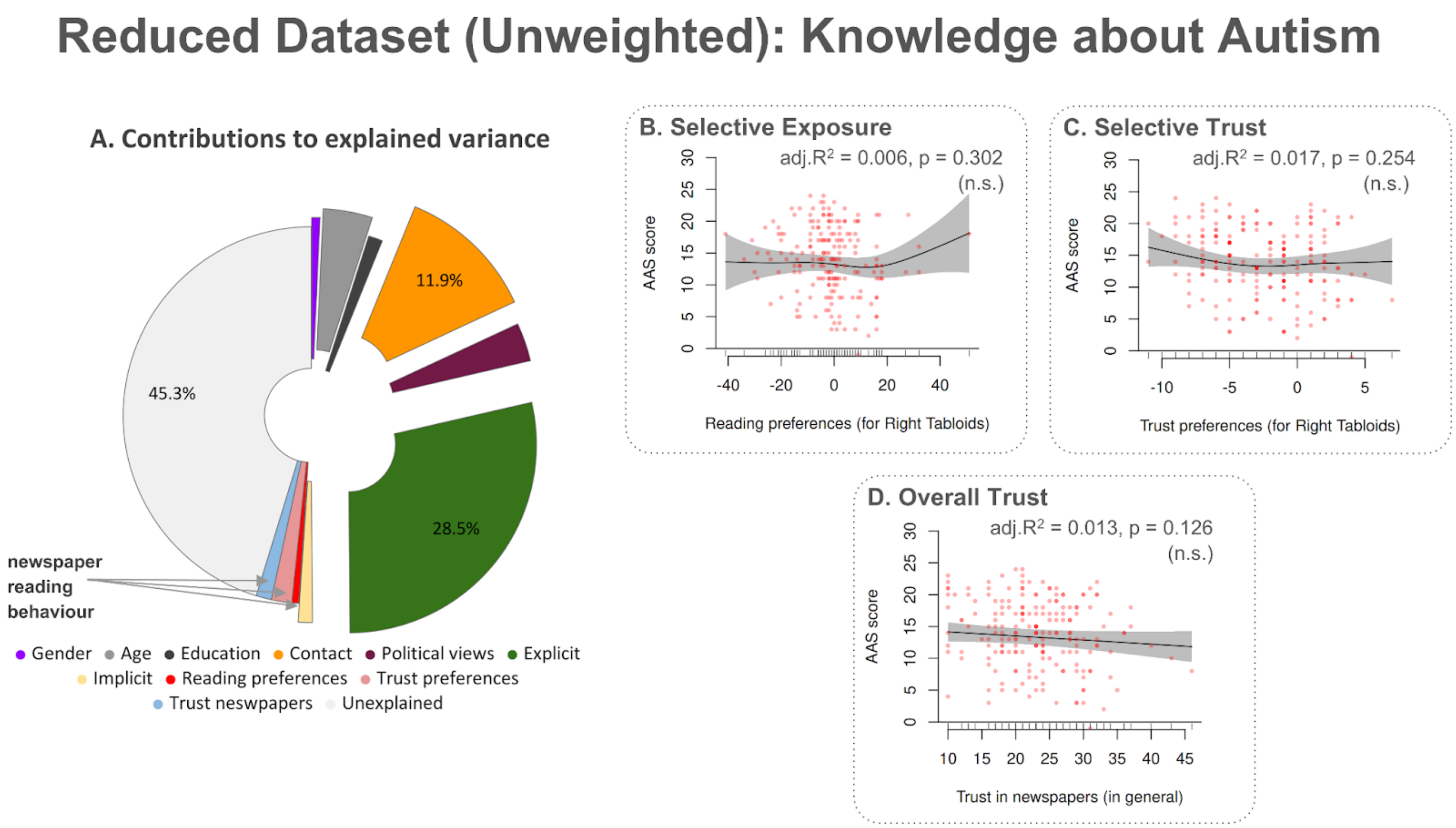

Supplementary Figure S13

A. The pie chart shows the contribution of various predictors to the explained variance in knowledge about autism.

B, C, and D. Panels illustrate the partial effects of three predictors related to newspaper reading preferences on knowledge about autism.

The configuration of this figure is identical to **Supplementary Figure S8**; the reader may refer to its caption for further details.

S16. Alternative weighted GAM model for Explicit attitudes: Eliminating factors with high collinearity/concurvity

Rationale/Relevance to the main paper

Hierarchical partitioning is an effective approach for handling collinearities or concurvities in the context of GAMs. However, as recommended by Lai et al. (2022, 2024) we conducted a supplementary analysis to examine potential high collinearities and suppressor effects by iteratively removing terms from the model that exhibited high concurvity, as measured with the *concurvity* function of the *mgcv* package (Wood, 2017), or negative individual contributions to explained variance based on the *gam.hp* package (Lai et al. 2024). We applied a threshold of 0.666 for the "worst concurvity" measure, corresponding to a Variance Inflation Factor (VIF) of at least 3, which is generally indicative of problematic multicollinearity.

This section presents results from the reduced model for **explicit attitudes**. We removed the selective trust variable as it had the highest concurvity value (worst concurvity = 0.99), likely driven by its close relationship with selective exposure (Spearman's Rho = 0.586, $p < 0.001$, as shown in **Supplementary Table S2**). The resulting model has acceptable concurvity for all terms and explains 60.7% of the variance in explicit attitudes, which is very similar to the model containing all terms (Figure 1 in the main paper).

In terms of effects, the reduced model still shows significant influences of gender, political orientation, contact with Autistic people, knowledge, and implicit attitudes, similar to the findings from the original model. Moreover, selective reading and overall trust in newspapers yield significant effects in this model. The selective reading effect exhibits an inverted U-shape suggesting that people with selective reading preferences for right-leaning tabloids tend to have more positive attitudes for the biggest range of values. We found a similar pattern in the original model..

Categorical Predictors

Supplementary Table S27. Estimates for Parametric coefficients (weighted GAM model for Explicit attitudes with a reduced number of factors to prevent concurvities).

	**Estimate**	**Std. Error**	**t value**	**p-value**
GENDERMale	1.5810	0.5235	3.020	0.00281 **
GENDEROther	2.1450	5.5218	0.388	0.69802

Note. *: p < .05, **: p < .01, ***: p < .001, Std. Error: Standard Error

Non-Linear Predictors

Supplementary Table S28. Estimates for Smooth terms (weighted GAM model for Explicit attitudes with a reduced number of factors to prevent concurvities).

Term	edf	Ref.edf	F	p-value
s(AGE)	2.340	2.705	3.414	0.03879 *
s(EDUCATION_YEARS)	1.001	1.001	6.073	0.01443 *
s(CONTACT)	1.000	1.001	17.100	4.97e-05 ***
s(POLITICAL)	2.742	2.935	17.100	0.00127 **
s(KNOWLEDGE)	4.641	5.769	32.015	< 2e-16 ***
s(IMPLICIT)	3.735	4.614	11.724	< 2e-16 ***
s(Read.Right.Tabloids)	2.716	3.447	1.493	0.21447
s(Trust.Newspapers.Amount)	5.254	6.462	2.628	0.02010 *

Note. *: $p < .05$, **: $p < .01$, ***: $p < .001$, edf (Estimated Degrees of Freedom): Represents the complexity of the smooth term. A value of 1 indicates an effectively linear effect, while higher values suggest increasing non-linearity. Ref. edf (Reference Degrees of Freedom): The maximum potential degrees of freedom that the smooth term could use, based on the basis dimension specified for the term in the model.

Individual Contributions and Effects of Reading behavior

Reduced factors: Explicit Attitudes

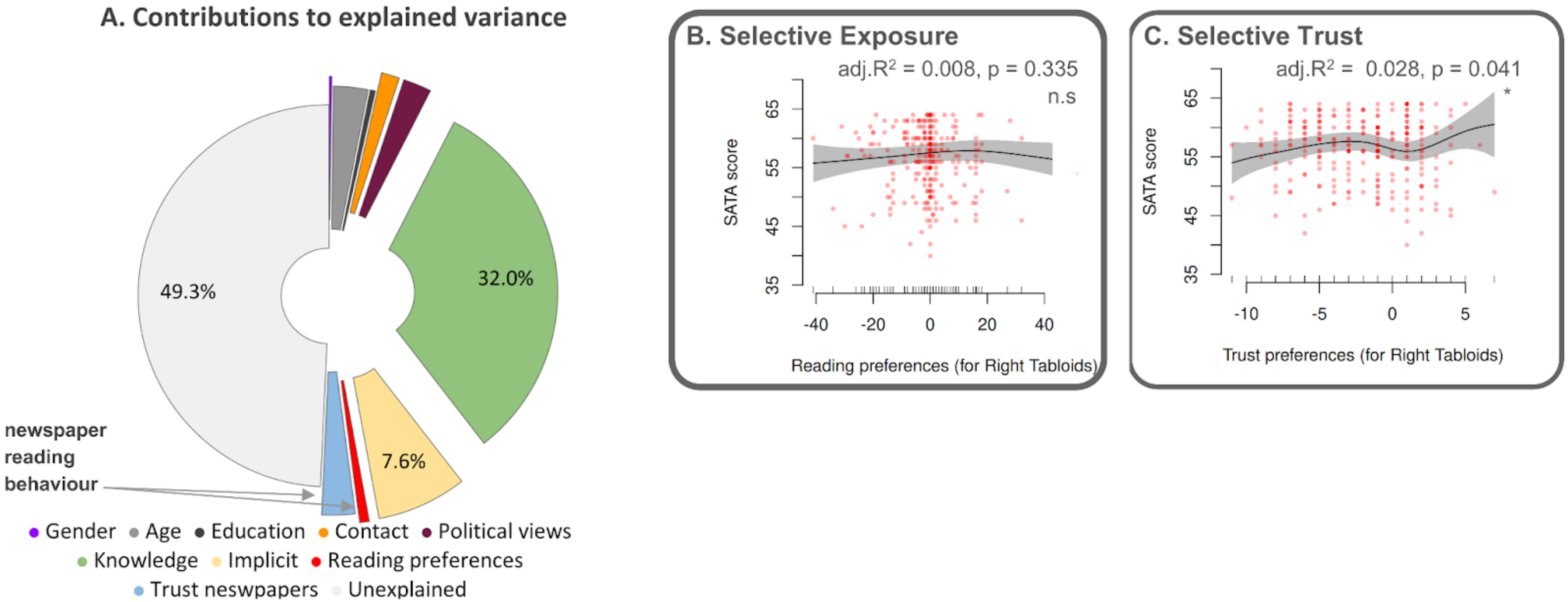

Supplementary Figure S14

A. The pie chart illustrates the contribution of various predictors to the explained variance in explicit attitudes toward autism, as measured by the Societal Attitudes Towards Autism Scale (SATA). We determined these contributions using hierarchical partitioning analysis for the GAM model weighted by overall newspaper exposure. This procedure calculated the proportion of adjusted R^2 attributed to individual predictors. The pie chart shows segments corresponding to different factors in the same order as the legend (starting at the top and moving clockwise). The distance of a segment from the centre indicates the significance of the corresponding factor (greater distance signifies lower p-values). The light gray segment represents variance that the GAM model left unexplained.

B, C, and D. These panels depict the partial effects of three predictors related to newspaper reading preferences on attitudes toward autism, based on estimates from the GAM model. **Panel B** shows the effect of preferences for right-leaning tabloids over left-leaning broadsheets, **Panel C** illustrates the effect of trust preferences for right-leaning tabloids, and **Panel D** presents the effect of overall trust in newspapers. The framing of each panel denotes the significance of the partial effects: a dotted line indicates a non-significant effect, a solid line signifies a significant effect, and a bold solid-line frame highlights a highly significant effect. The individual contribution in adjusted R^2 and the corresponding p-values (*: $p < .05$, **: $p < .01$, ***: $p < .001$) are shown at the top of each plot.

S17. Alternative GAM model for Implicit attitudes: : Eliminating factors with high collinearity/concurvity

Rationale/Relevance to the main paper

Again, as recommended by Lai et al.,[6, 7] we conducted a supplementary analysis to address potential high concurvities and suppressor effects. Specifically, we iteratively removed terms that demonstrated high concurvity or negative individual contributions to explained variance in the GAM model for **implicit attitudes.**

This section presents the results from the reduced model, where the selective trust variable again due to a "worst concurvity" value of 0.99 (stemming from its close relationship with selective exposure; Spearman's Rho = 0.586, $p < 0.001$). Furthermore, we removed Gender because it contributed negatively to the adjusted R^2. The final model explains 34.2% of the variance in explicit attitudes, which is very similar to the model that included all terms.

In terms of effects, this reduced model yields significant effects of knowledge, contact, and explicit attitudes, which are comparable to the findings of the original model. Selective reading and overall trust in newspapers also remain significant. **The shape of the selective reading effect is practically identical to that observed in the original model, indicating that individuals with greater exposure to right-leaning tabloids tend to have more negative attitudes. Thus, this model replicates the effect detected in the other models.** However, the effect of overall trust remains difficult to interpret due to a highly variable ("wiggly") partial effect function.

Categorical Predictors

Supplementary Table S29. Estimates for Parametric coefficients (weighted GAM model for Implicit attitudes with a reduced number of factors to prevent concurvities).

	**Estimate**	**Std. Error**	**t value**	**p-value**
GENDERMale	-0.06968	0.04137	-1.684	0.0935 .
GENDEROther	-0.29634	0.42705	-0.694	0.4884

Note. *: p < .05, **: p < .01, ***: p < .001, Std. Error: Standard Error

Non-Linear Predictors

Supplementary Table S30. Estimates for Smooth terms (weighted GAM model for Implicit attitudes with a reduced number of factors to prevent concurvities)***.***

Term	edf	Ref.edf	F	p-value
s(AGE)	2.270	2.638	2.443	0.1232
s(EDUCATION_YEARS)	2.030	2.403	2.207	0.1057
s(CONTACT)	1.001	1.001	23.572	2.52e-06 ***
s(POLITICAL)	2.522	2.815	3.072	0.0235 *
s(KNOWLEDGE)	5.795	7.083	5.144	1.80e-05 ***
s(EXPLICIT)	4.800	5.905	8.618	< 2e-16 ***
s(Read.Right.Tabloids)	1.000	1.000	9.971	0.0018 **
s(Trust.Newspapers.Amount)	6.881	8.172	2.420	0.0142 *

Note. *: p < .05, **: p < .01, ***: p < .001, edf (Estimated Degrees of Freedom): Represents the complexity of the smooth term. A value of 1 indicates an effectively linear effect, while higher values suggest increasing non-linearity. Ref. edf (Reference Degrees of Freedom): The maximum potential degrees of freedom that the smooth term could use, based on the basis dimension specified for the term in the model.

Individual Contributions and Effects of Reading behavior

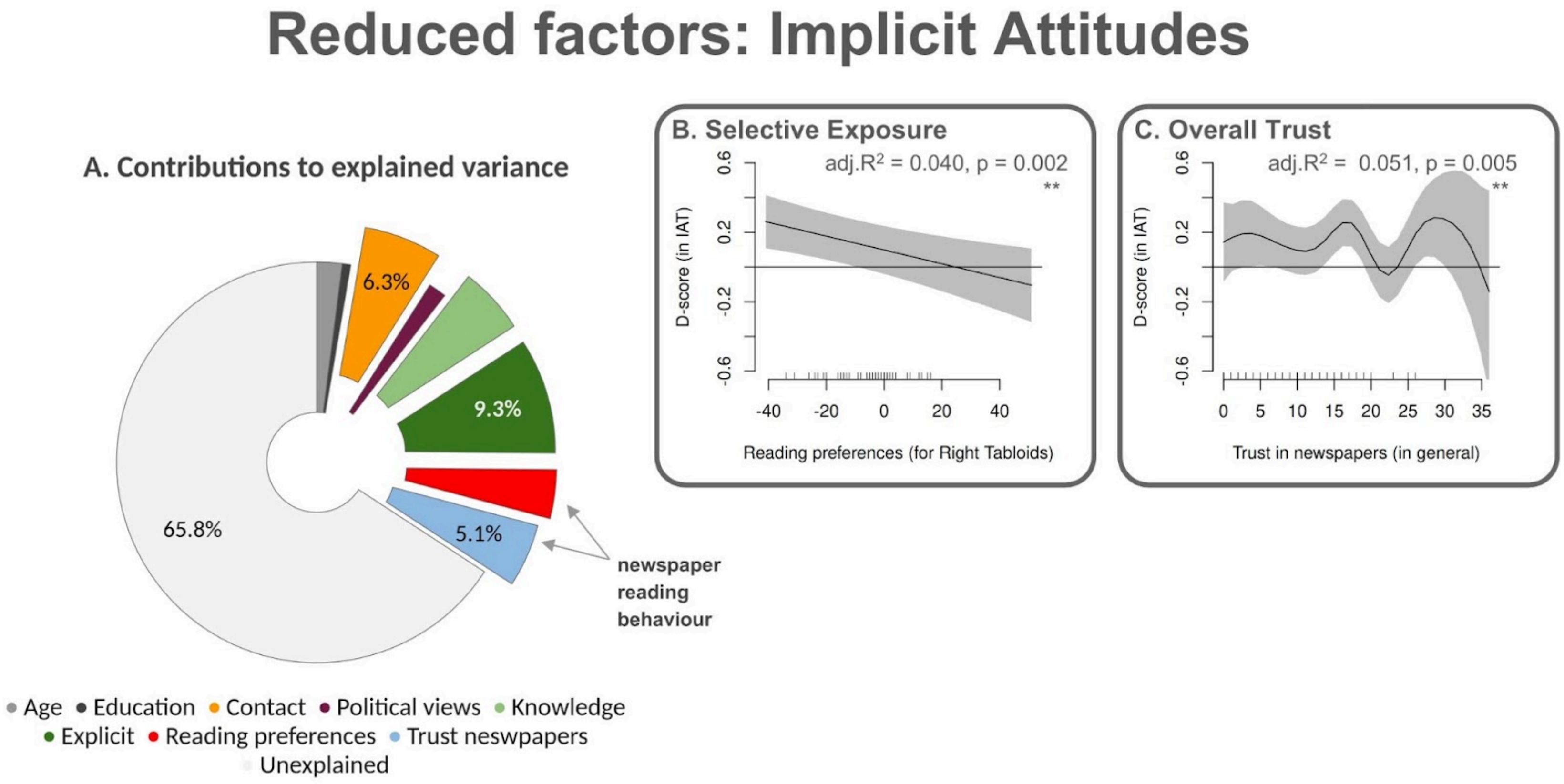

Supplementary Figure S15

A. The pie chart illustrates the contribution of various predictors to the explained variance in implicit attitudes toward autism, measured using the AAS.

B, C, and D. Panels depict the partial effects of three predictors related to newspaper reading preferences on implicit attitudes.

The configuration of this figure is identical to ***Supplementary Figure S11***; the reader may refer to its caption for further details.

S18. Alternative GAM model for Knowledge about Autism: : Eliminating factors with high collinearity/concurvity

Rationale/Relevance to the main paper

Again, as recommended by Lai et al. (2022, 2024) we conducted a supplementary analysis to address potential high concurvities and suppressor effects. Specifically, we iteratively removed terms that demonstrated high concurvity or negative individual contributions to explained variance in the GAM model for Knowledge about Autism.

This section presents the results from the reduced model, where, again, we removed the selective trust variable due to a “worst concurvity” value of 0.99, which stemmed from its close relationship with selective exposure (Spearman’s Rho = 0.586, $p < 0.001$). This reduced model explains 72.1% of the variance in explicit attitudes, which is very similar to the model that included all terms.

In terms of effects, the reduced model retains significant predictors for gender, age, political bias, contact, and both explicit and implicit attitudes, consistent with the original model. Furthermore, selective reading and overall trust in newspapers remain significant predictors in this reduced-factors model. The effect of selective reading is nearly identical in shape to that observed in the original model: individuals with greater exposure to right-leaning tabloids exhibit slightly less knowledge about autism, with some tendency for knowledge to increase beyond a certain threshold. Additionally, the model replicates the effect of overall trust in newspapers on knowledge about autism, whereby individuals who trust newspapers more tend to have less accurate knowledge about autism. Overall the results of this reduced model are highly similar with the original model (**Figure 3**).

Categorical Predictors

Supplementary Table S31. Estimates for Parametric coefficients (weighted GAM model for Knowledge about Autism with a reduced number of factors to prevent concurvities).

	**Estimate**	**Std. Error**	**t value**	**p-value**
GENDERMale	-1.3276	0.3783	-3.509	0.000538 ***
GENDEROther	-4.1822	4.2118	-0.993	0.321743

Note. *: p < .05, **: p < .01, ***: p < .001, Std. Error: Standard Error

Non-Linear Predictors

Supplementary Table S32. Estimates for Smooth terms (weighted GAM model for Knowledge about Autism with a reduced number of factors to prevent concurvities)..

Term	edf	Ref.edf	F	p-value
s(AGE)	1.000	1.000	14.556	0.000174 ***
s(EDUCATION_YEARS)	1.000	1.000	7.790	0.005682 **
s(CONTACT)	2.130	2.500	37.719	< 2e-16 ***
s(POLITICAL)	2.946	2.996	26.934	< 2e-16 ***
s(EXPLICIT)	1.984	2.497	70.195	< 2e-16 ***
s(IMPLICIT)	3.204	3.991	7.239	1.79e-05 ***
s(Read.Right.Tabloids)	4.070	4.996	5.338	0.000114 ***
s(Trust.Newspapers.Amount)	6.257	7.503	3.656	0.000713 ***

Note. *: $p < .05$, **: $p < .01$, ***: $p < .001$, edf (Estimated Degrees of Freedom): Represents the complexity of the smooth term. A value of 1 indicates an effectively linear effect, while higher values suggest increasing non-linearity. Ref. edf (Reference Degrees of Freedom): The maximum potential degrees of freedom that the smooth term could use, based on the basis dimension specified for the term in the model.

Individual Contributions and Effects of Reading behavior

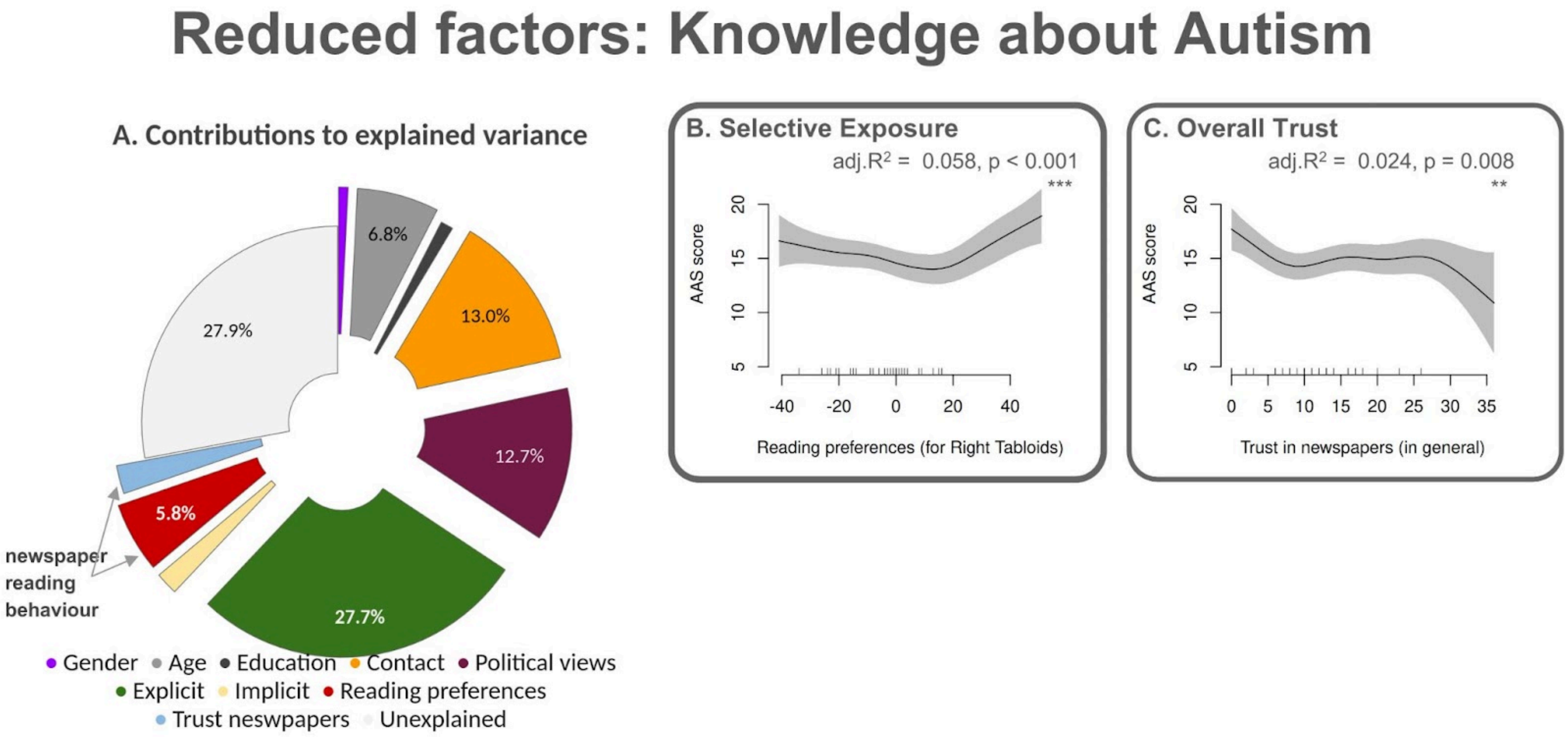

Supplementary Figure S16

A. The pie chart shows the contribution of various predictors to the explained variance in knowledge about autism.

B, C, and D, E. Panels illustrate the partial effects of three predictors related to newspaper reading preferences on knowledge about autism.

The configuration of this figure is identical to ***Supplementary Figure S11***; the reader may refer to its caption for further details.

References

Carpenter, T. P., Pogacar, R., Pullig, C., Kouril, M., Aguilar, S., LaBouff, J., Isenberg, N., & Chakroff, A. (2019). Survey-software implicit association tests: A methodological and empirical analysis. *Behavior Research Methods, 51*(5), 2194–2208. https://doi.org/10.3758/s13428-019-01293-3

Greenwald, A. G., McGhee, D. E., & Schwartz, J. L. K. (1998). Measuring individual differences in implicit cognition: The implicit association test. *Journal of Personality and Social Psychology, 74*(6), 1464–1480. https://doi.org/10.1037/0022-3514.74.6.1464

Flood, L. N., Bulgrin, A., & Morgan, B. L. (2013). Piecing together the puzzle: Development of the Societal Attitudes towards Autism (SATA) scale. *Journal of Research in Special Educational Needs, 13*(2), 121–128. https://doi.org/10.1111/j.1471-3802.2011.01224.x

Tipton, L. A., & Blacher, J. (2014). Brief report: Autism awareness: Views from a campus community. *Journal of Autism and Developmental Disorders, 44*(2), 477–483. https://doi.org/10.1007/s10803-013-1893-9

Gillespie-Lynch, K., Brooks, P. J., Someki, F., Obeid, R., Shane-Simpson, C., Kapp, S. K., & Smith, D. S. (2015). Changing college students' conceptions of autism: An online training to increase knowledge and decrease stigma. *Journal of Autism and Developmental Disorders, 45*(8), 2553–2566. https://doi.org/10.1007/s10803-015-2422-9

Lai, J., Tang, J., Li, T., Zhang, A., & Mao, L. (2024). Evaluating the relative importance of predictors in Generalized Additive Models using the gam.hp R package. *Plant Diversity, 46*(4), 542–546. https://doi.org/10.1016/j.pld.2024.06.002

Lai, J., Zou, Y., Zhang, J., & Peres-Neto, P. (2022). Generalizing hierarchical and variation partitioning in multiple regression and canonical analyses using the rdacca.hp R package. *Methods in Ecology and Evolution, 13*(4), 782–788. https://doi.org/10.1111/2041-210X.13800

Wood, S. N. (2017). *Generalized additive models: An introduction with R* (2nd ed.). Chapman and Hall/CRC.
